# γδ T Cells Confer Protection against Murine Cytomegalovirus (MCMV)

**DOI:** 10.1371/journal.ppat.1004702

**Published:** 2015-03-06

**Authors:** Camille Khairallah, Sonia Netzer, Arnaud Villacreces, Marina Juzan, Benoît Rousseau, Sara Dulanto, Alban Giese, Pierre Costet, Vincent Praloran, Jean-François Moreau, Pierre Dubus, David Vermijlen, Julie Déchanet-Merville, Myriam Capone

**Affiliations:** 1 Université de Bordeaux, Bordeaux, France; 2 CNRS, UMR 5164, Bordeaux, France; 3 Laboratoire d’Immunologie et d’Immunogénétique, Animalerie A2, Bordeaux, France; 4 Faculty of Pharmacy, Université Libre de Bruxelles (ULB), Brussels, Belgium; 5 EA2406 Histologie et pathologie moléculaire des tumeurs, Bordeaux, France; 6 Animalerie spécialisée, Bordeaux, France; 7 Laboratoire d’Hématologie, Centre Hospitalo-Universitaire, Bordeaux, France; 8 Centre Hospitalo-Universitaire, Bordeaux, France; 9 Institute for Medical Immunology, Université Libre de Bruxelles (ULB), Brussels, Belgium; University of Minnesota Medical School, UNITED STATES; University of Southern California School of Medicine, UNITED STATES

## Abstract

Cytomegalovirus (CMV) is a leading infectious cause of morbidity in immune-compromised patients. γδ T cells have been involved in the response to CMV but their role in protection has not been firmly established and their dependency on other lymphocytes has not been addressed. Using C57BL/6 αβ and/or γδ T cell-deficient mice, we here show that γδ T cells are as competent as αβ T cells to protect mice from CMV-induced death. γδ T cell-mediated protection involved control of viral load and prevented organ damage. γδ T cell recovery by bone marrow transplant or adoptive transfer experiments rescued CD3ε^−/−^ mice from CMV-induced death confirming the protective antiviral role of γδ T cells. As observed in humans, different γδ T cell subsets were induced upon CMV challenge, which differentiated into effector memory cells. This response was observed in the liver and lungs and implicated both CD27^+^ and CD27^−^ γδ T cells. NK cells were the largely preponderant producers of IFNγ and cytotoxic granules throughout the infection, suggesting that the protective role of γδ T cells did not principally rely on either of these two functions. Finally, γδ T cells were strikingly sufficient to fully protect Rag^−/−^γc^−/−^ mice from death, demonstrating that they can act in the absence of B and NK cells. Altogether our results uncover an autonomous protective antiviral function of γδ T cells, and open new perspectives for the characterization of a non classical mode of action which should foster the design of new γδ T cell based therapies, especially useful in αβ T cell compromised patients.

## Author Summary

γδ T cells are unconventional T lymphocytes that play a unique role in host protection against pathogens. Human Cytomegalovirus (HCMV) is a widespread virus that can cause severe organ disease such as hepatitis and pneumonitis in immune-compromised patients. Our decade-long study conveys compelling evidence for the implication of human γδ T cells in the immune response against HCMV, but their protective role could not be formally demonstrated in humans. In the present study we use the murine model of CMV infection which allows the spatial and temporal analysis of viral spread and anti-viral immune responses. We show that, in the absence of αβ T cells, γδ T cells control MCMV-induced hepatitis, pneumonitis and death by restricting viral load in the liver, lungs and spleen. γδ T cells expand in these organs and display memory features that could be further incorporated into vaccination strategies. In conclusion, γδ T cells represent an important arm in the immune response against CMV infection that could be particularly important in the context of αβ T cell immune-suppression.

## Introduction

Human CMV (HCMV) is a universally distributed pathogen that infects 50–90% of the world's population. Asymptomatic in healthy people, HCMV infection may lead to increased morbidity and mortality in immunocompromised individuals. Overall survival following transplantation is decreased when either the donor or the recipient is HCMV-seropositive [1,2,3]. Because of drug-related adverse effects and drug resistance there is growing interest for immunotherapy as an adjunct to antiviral therapy. Understanding the mechanisms developed by the immune system to control HCMV is therefore critical to enable the design of new curative or preemptive protocols aimed at enhancing patient immune defense against this virus.

Effective immune control of HCMV has been compellingly shown to rely on both conventional lymphocytes and NK cells [4]. However, as we initially reported, HCMV also induces a robust γδ T cell response in organ transplant recipients [5]; and later, γδ T cell response to HCMV was extended to several other situations not always associated to immunosuppression; such as immunodeficiencies, bone marrow transplantation, pregnancy, elderly and also in healthy individuals [6,7,8,9,10,11,12]. HCMV-mediated persistent expansion of γδ T cells in transplant recipients is associated with infection resolution [13], and implies tissue-associated Vδ2-negative γδ T cells which acquire a terminally differentiated phenotype upon HCMV pressure [10,14]. When isolated *in vitro*, these lymphocytes were shown to kill HCMV-infected cells, limit virus propagation and produce IFNγ through recognition of opsonized viruses [15,16].

Several features of γδ T cells might explain their specific relationship to HCMV: (i) they are not MHC restricted, and thus not affected by HCMV strategies to inhibit HLA molecules, (ii) they recognize self-antigens on the surface of stressed cells such as virus infected cells [17,18] and (iii) they are located at external body surfaces (eg gut and lung) and organs (eg liver) involved in HCMV transmission and replication [19]. Moreover, HCMV-reactive γδ T cells exhibit dual reactivity against tumor cells, due to the recognition of stress-induced self-antigens shared by HCMV-infected and tumor cells [15,18,20]. In agreement with this, HCMV-infection and/or γδ T cell expansion have been associated with reduced cancer risk in kidney transplant recipients [21] and with graft-versus leukemia effect in bone marrow transplant recipients [22,23,24].

All these specificities are consistent with an antiviral protective role of γδ T cells against HCMV and they thus represent valuable candidates for anti-HCMV immunotherapy especially in immunocompromised patients vulnerable to neoplasia. However, their role in protection and specific contribution within the global anti-CMV immune response has not been firmly established, nor their anatomical sites of activation and intervention. The aim of the present study was therefore to take advantage of the murine model of CMV infection to address these questions and to assess the respective ability of αβ and γδ T cells alone to protect mice from CMV infection. Murine CMV (MCMV) has been widely used to model the immune response to HCMV in mice since it reproduces with reasonable accuracy the antiviral response of CD8 T cells and NK cells [25]. Murine γδ T cells have been implicated in MCMV infection only once [26], and their sufficiency for protection has not yet been addressed. We show herein that γδ T cells are as competent as αβ T cells to control MCMV infection and protect mice from death encouraging the development of novel anti-viral immunotherapeutic protocols based on γδ T cell manipulation.

## Results

### γδ T cells are as efficient as αβ T cells to protect mice from MCMV-induced death

**Fig 1:**
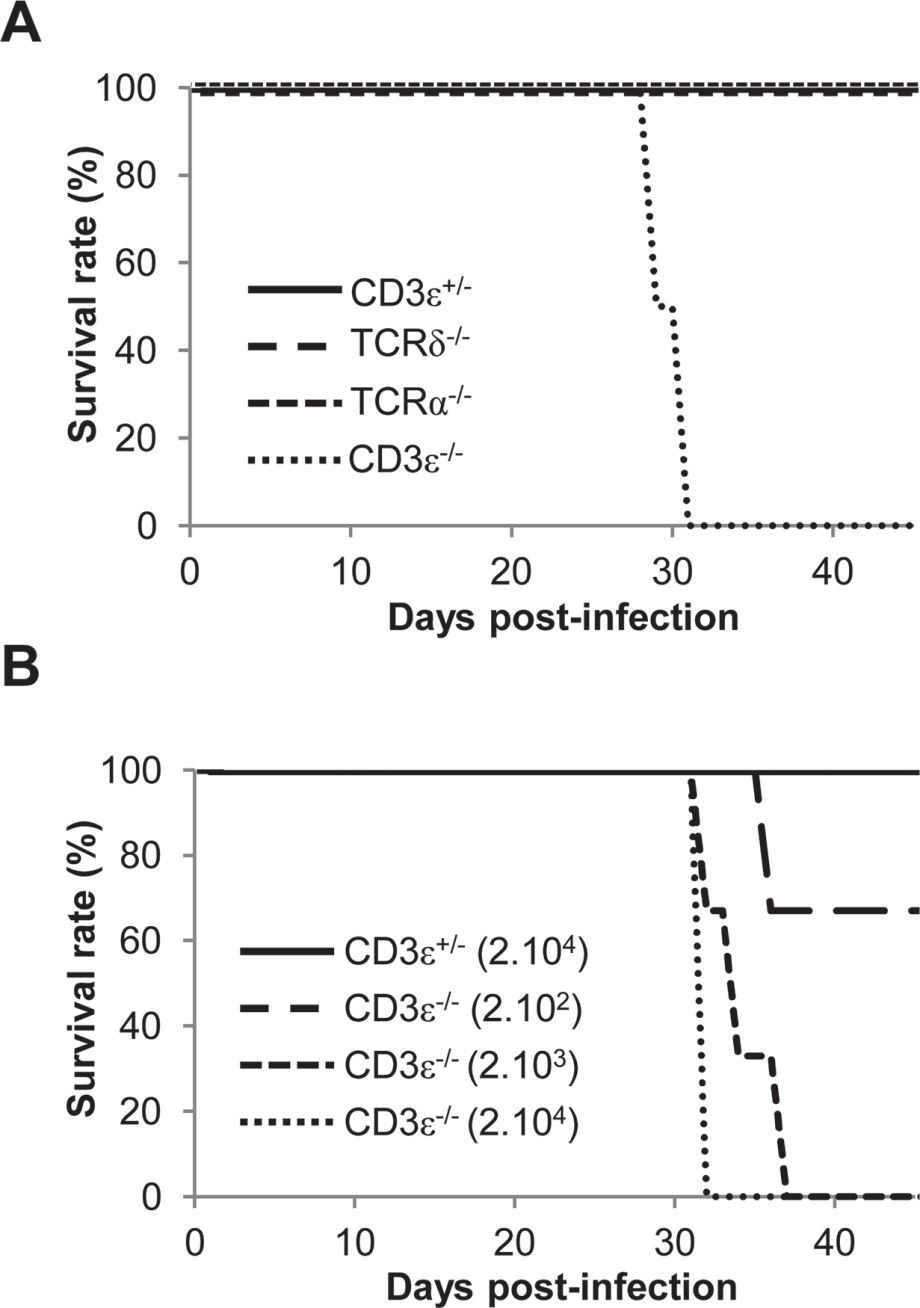
γδ T cells prevent mice from MCMV-induced mortality. **A.** TCRδ^−/−^, TCRα^−/−^ CD3ε^+/−^ and CD3ε^−/−^ mice (10 of each) were infected i.p. with 1.10^5^ PFU of MCMV at day 0 and monitored every other day for mortality. Data are from one representative of 3 independent experiments. **B.** CD3ε^+/−^ and CD3ε^−/−^ mice (4 of each) were infected i.p. with indicated doses of MCMV at day 0 and monitored every day for mortality. Data are from one experiment.

In mice, MCMV-specific αβ T cells control viral spread and protect infected mice from death [27] but little is known regarding the implication of γδ T cells. To evaluate the respective contribution of αβ and γδ T cells to the immune response against MCMV, mice deficient for γδ T cells (TCRδ^−/−^), for αβ T cells (TCRα^−/−^) or for both T cell subsets (CD3ε^−/−^) were challenged with 10^5^ plaque forming units (PFU) of salivary gland MCMV. This dose was reported to be sublethal for C57BL/6 mice (as described at http://mutagenetix.utsouthwestern.edu/protocol/protocol_rec.cfm?protocolid=5). Accordingly, 100% of CD3ε^+/−^ control mice survived MCMV infection, whereas CD3ε^−/−^ died about 4 weeks after viral challenge (Fig. 1A), confirming the critical role of T cells in controlling MCMV infection. CD3ε^−/−^ mice were extremely sensitive to MCMV despite the presence of NK cells [28] since they died at doses of MCMV as low as 2.10^3^ PFU (Fig. 1B). Unexpectedly, both TCRδ^−/−^ and TCRα^−/−^ mice survived as long as CD3ε^+/−^ control mice. These results reveal that the presence of either αβ or γδ T cell subset was sufficient to protect mice from MCMV infection, disclosing the potentially critical function of γδ T cells in the immune response against MCMV.

### γδ T cells control viral loads in organs

**Fig 2:**
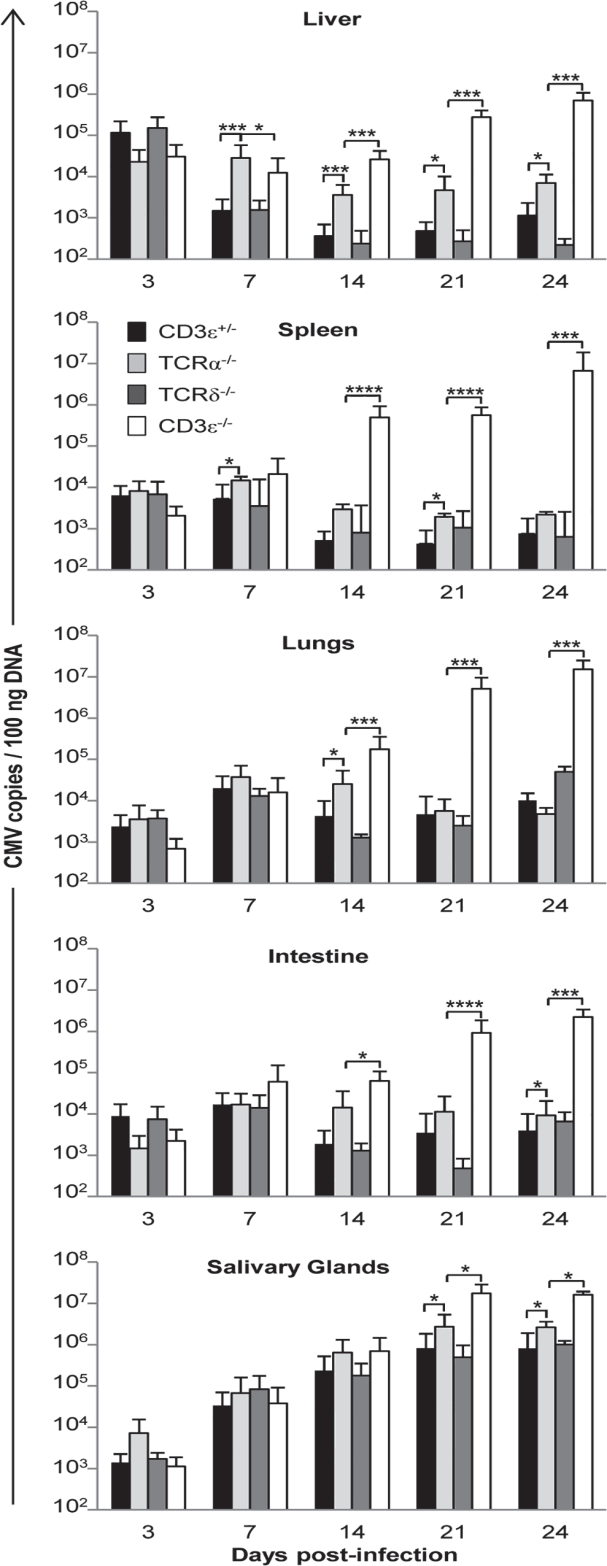
MCMV dissemination in lungs, spleen, liver, intestine and salivary glands from T cell competent and T cell deficient mice. TCRδ^−/−^, TCRα^−/−^, CD3ε^+/−^, and CD3ε^−/−^ mice were infected i.p. with 2.10^3^ PFU of MCMV. At indicated days post-infection, 4 mice of each mouse line were dissected and MCMV gB was quantified in organs by real time PCR. The experiment was repeated 3 times under similar conditions. Histograms represent means of MCMV DNA copy number (per 100 ng genomic DNA) ± SD of all mice from the three experiments (n = 4x3 mice). Statistical differences between viral loads in TCRα^−/−^ versus CD3ε^+/−^ mice, and in TCRα^−/−^ versus CD3ε^−/−^ mice are shown.

To examine whether this protection against CMV by γδ T cells relies on the control of viral loads, the kinetics of MCMV spread in T cell deficient versus T cell competent mice was determined in various organs. Comparison between each mouse line is shown in Fig. 2 and comparison between different time points is shown in S1 Fig. In the absence of T cells, MCMV DNA copy numbers increased substantially from day 3 to 24, with up to 10^7^ copies (/100ng DNA) in the spleen and lungs of CD3ε^−/−^ mice before death. Interestingly, γδ T cells alone (in TCRα^−/−^ mice) were sufficient to prevent an increase of viral load in all organs, except the salivary glands which are known to support prolonged virus replication even in wild-type mice (S1 Fig.). At the end of these experiments, MCMV copies were much lower in T cell bearing mice than in mice without T cells (Fig. 2), underlining the inability of C57BL/6 mice to control MCMV infection in the absence of T cells. It was of particular interest to see that in the lungs γδ T cells were as potent as αβ T cells to control the viral load except at day 14. As a whole, these results suggest independent control of MCMV spread by the αβ and γδ T cell subsets, revealing that γδ T cells are sufficient to control viral load and can substitute for the absence of αβ T cells.

### γδ T cell-dependent control of viral load associates with reduced organ damage

**Fig 3:**
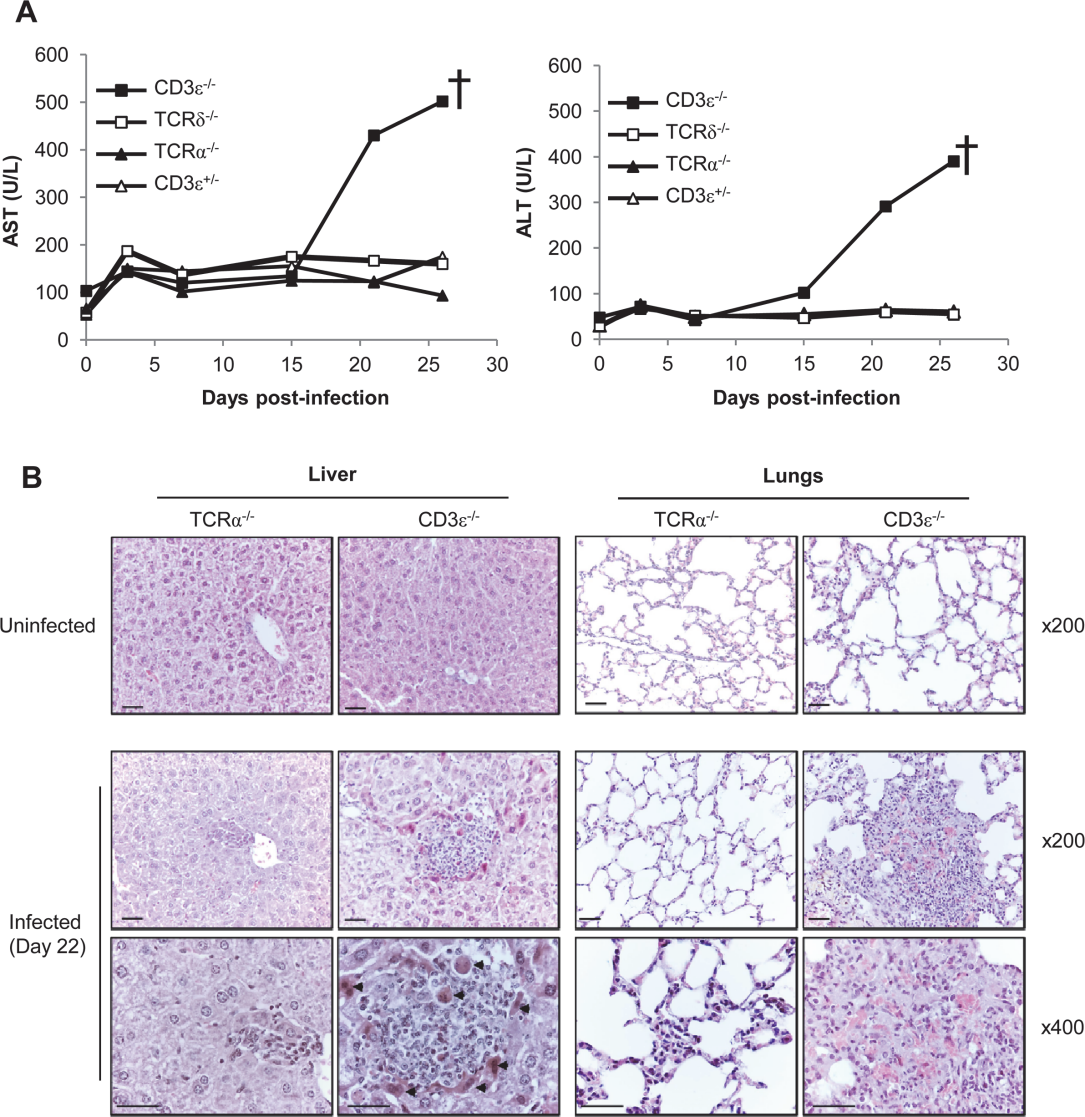
γδ T cell control of MCMV infection is associated with reduced organ damage. **A.** TCRδ^−/−^, TCRα^−/−^, CD3ε^+/−^ and CD3ε^−/−^ mice were infected i.p. as indicated in Fig. 1A. 3 mice/group were bled at days 0, 3, 7, 15, 21 and just before death for biochemical analyses of AST and ALT in serums. The experiment was repeated twice and data obtained for one representative mouse/group are shown. † death of CD3ε^−/−^. **B.** TCRα^−/−^ and CD3ε^−/−^ mice were uninfected, or i.p. infected with 2.10^3^ pfu of MCMV. Uninfected and Day 22-infected mice were sacrificed and the liver and lungs were embedded in paraffin for HES staining. Apoptotic hepatocytes are shown (arrowheads). Scale bar = 200 mm. Magnifications are indicated in the right-hand side of the figure. The data are from one representative of 3 mice for each condition.

Hepatitis and pneumonitis are common features of CMV pathogenesis in both humans and mice. Hepatitis can be assessed in living infected mice through the quantification of transaminase levels in the serum. As shown in Fig. 3A, aspartate aminotransferase (AST) and alanine aminotransferase (ALT) only increased in the absence of all T cells (CD3ε^−/−^ mice), reaching up to 8 fold the basal level before death of CD3ε^−/−^ mice. Accordingly, histological analysis of livers from CD3ε^−/−^ infected mice before death (day 22) showed typical features of active hepatitis, with many large granulomas mainly composed of histiocytic cells associated with multiple apoptotic hepatocytes (Fig. 3B). In contrast, only a few small granulomas were observed in TCRα^−/−^ mice livers at that time point. Furthermore, CD3ε^−/−^ mice presented an active pneumopathy with large granulomas and hemorrhagic foci at day 22, while TCRα^−/−^ lung histology was close to normal with only a slight increase of inflammatory cells in the inter-alveolar septa (Fig. 3B). In conclusion, CD3ε^−/−^ mice showed clear evidences of both liver and lung diseases 3 weeks post MCMV infection, in agreement with the high viral loads found at that time in these organs. In contrast, liver and lung disorders were not observed in TCRα^−/−^ mice, emphasizing the ability of γδ T cells to control MCMV infection and associated organ disease. Whether γδ T cells limit organ disease only as a consequence of viral replication control or also by producing mediators of tissue repair deserves further attention.

### Expansion of γδ T cells in the liver and lungs of MCMV-infected TCRα^−/−^ mice

**Fig 4:**
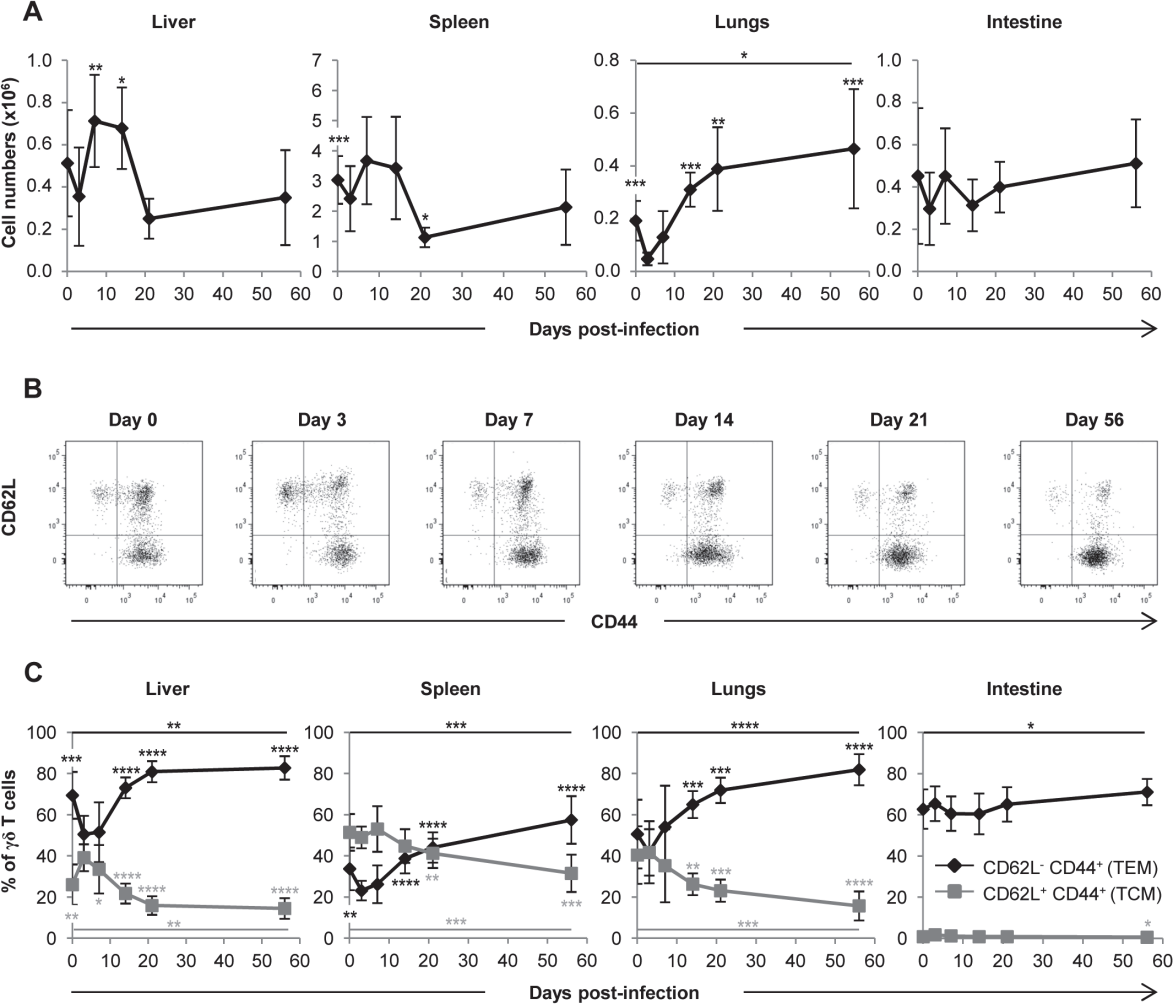
Mobilization of γδ T cells in MCMV-infected organs from TCRα^−/−^ mice. TCRα^−/−^ mice were infected i.p. with 2.10^3^ PFU of MCMV. At indicated post-infection days, 5–9 mice were sacrificed, immune cells prepared from each organ and γδ T cells stained as shown in S2 Fig. **A.** Kinetics of absolute γδ T cell numbers determined as described in methods. Presented data are mean ± SEM of 8–9 mice from one representative of 2 experiments. **B**. CD62L and CD44 expression by lymphocytes was evaluated by flow cytometry, with the presented gating strategy (lungs shown as example). **C.** Longitudinal analysis of γδ T cell phenotype in all organs. Results are pooled from 2 independent experiments representing a total of 13–14 mice (means ± SEM). Statistical differences of cell numbers and percentages between day 3 and other time points are shown, as well as statistical differences between days 0 and 56 (solid line).

We next sought to analyze whether the control of MCMV spread was associated with an amplification of γδ T cells in infected organs. S2 Fig. shows the gating strategy used for γδ T cell flow cytometry analysis. After a slight decrease at day 3, γδ T cell numbers increased importantly in the lungs until day 21 (approximately 8 fold), and this rise persisted until the end of the experiment. A significant but more modest and transient increase was also observed in the liver (approximately 2 fold from day 3 to 7). By contrast and to our surprise given their preponderance in gut intraepithelial lymphocytes, no significant variation of γδ T cells was observed in the intestine. In the spleen, γδ T cells levels remained stable until day 21 when they decreased (Fig. 4A). In conclusion, control of MCMV infection by γδ T cells in TCRα^−/−^ mice is associated with a transient γδ T cell increase in the liver, and a delayed but strong and persistent expansion of γδ T cells in the lungs.

### γδ T cells responding to MCMV display an effector-memory phenotype

We next asked whether γδ T cells responding to MCMV differentiate into effector-memory cells as we observed previously in humans [10,14]. After a transient decrease early post MCMV challenge, the proportion of effector memory (EM, CD44^+^CD62L^−^) γδ T cells increased in the spleen, liver and lungs concomitantly with a decrease of central memory (CM, CD44^+^CD62L^+^) γδ T cells. Effector memory γδ T cells reached more than 80% in the liver and lungs at day 56 (Fig. 4B and 4C). Consistent with the absence of variation in γδ T cell numbers in the intestine, no modification of γδ T cells phenotype could be observed in this organ. These results confirm that MCMV induces a marked response of γδ T cells in the lungs and liver, which is more modestly seen in the spleen and absent from the intestine.

### Vγ1^+^ and Vγ4^+^ γδ T cell subsets are both involved in the response to MCMV

**Fig 5:**
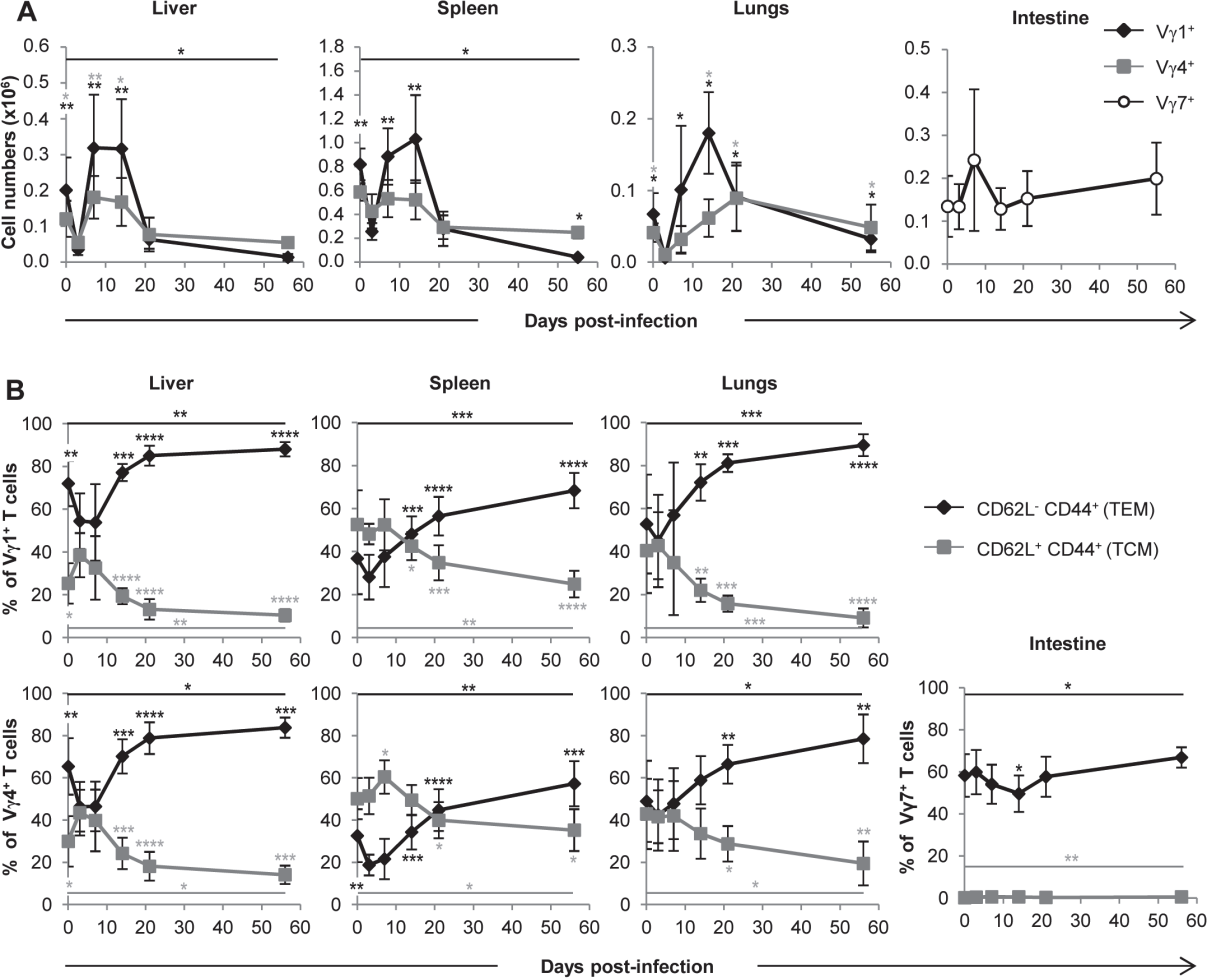
Both Vγ1 and Vγ4 subset are involved in γδ T cell response to MCMV. TCRα^−/−^ mice were infected i.p. with 2.10^3^ PFU of MCMV. At indicated days post-infection, 5–9 mice were sacrificed and immune cells were prepared from each organ. Expression of Vγ1, Vγ4 and Vγ7 chains by lymphocytes was evaluated by flow cytometry (S2 Fig.). **A.** Kinetics of absolute cell numbers for each subset. Presented data are mean ± SEM of 8–9 mice from one representative of 2 experiments. **B**. CD62L and CD44 expression by γδ T cell subsets was evaluated by flow cytometry in all organs. Results are pooled from 2 independent experiments representing a total of 13–14 mice (means ± SEM). Statistical differences of cell numbers and percentages between day 3 and other time points are shown, as well as statistical differences between days 0 and 56 (solid line).

The subsets of murine γδ T lymphocytes expressing the Vγ1 or Vγ4 chains of the TCR predominate in the spleen, liver and lungs, whereas intestinal γδ T cells are almost exclusively Vγ7^+^ (nomenclature of Heilig and Tonegawa [29]). We assessed the quantity, repertoire and memory phenotype of these γδ T lymphocyte subsets in the liver, spleen and lungs. Not surprisingly, low proportions of Vγ1^+^ γδ T cells were found in the intestine (S2 Fig.). As observed in Fig. 5A, the expansion of γδ T cells in the lungs and liver after day 3 concerned mainly Vγ1^+^ but also Vγ4^+^ γδ T cells. Both subsets followed the kinetics of total γδ T cells (Fig. 4A). Analysis of subsets also showed a response of Vγ1^+^, but not Vγ4^+^ T cells, in the spleen (Fig. 4A and Fig. 5A). The proportion of EM cells among both Vγ1^+^ and Vγ4^+^ γδ T cells increased after day 3 in the lungs, liver and spleen (Fig. 5B). In contrast, Vγ7^+^ γδ T cell numbers/memory phenotype did not vary significantly upon MCMV infection (Fig. 5A and Fig. 5B), as could be expected from the analysis of the whole γδ T cell population in the intestine (Fig. 4A and Fig. 4C). The complementary-determining-region (CDR3)γ1 and CDR3γ4 length profile of liver, spleen and lung-derived γδ T cells were not different between uninfected and infected mice for 14 days (S3 Fig. and S4 Fig.), indicating that there were no major changes in these CDR3 repertoires after expansion.

### γδ T cells recovery rescues CD3ε^−/−^ mice from MCMV-induced death

**Fig 6:**
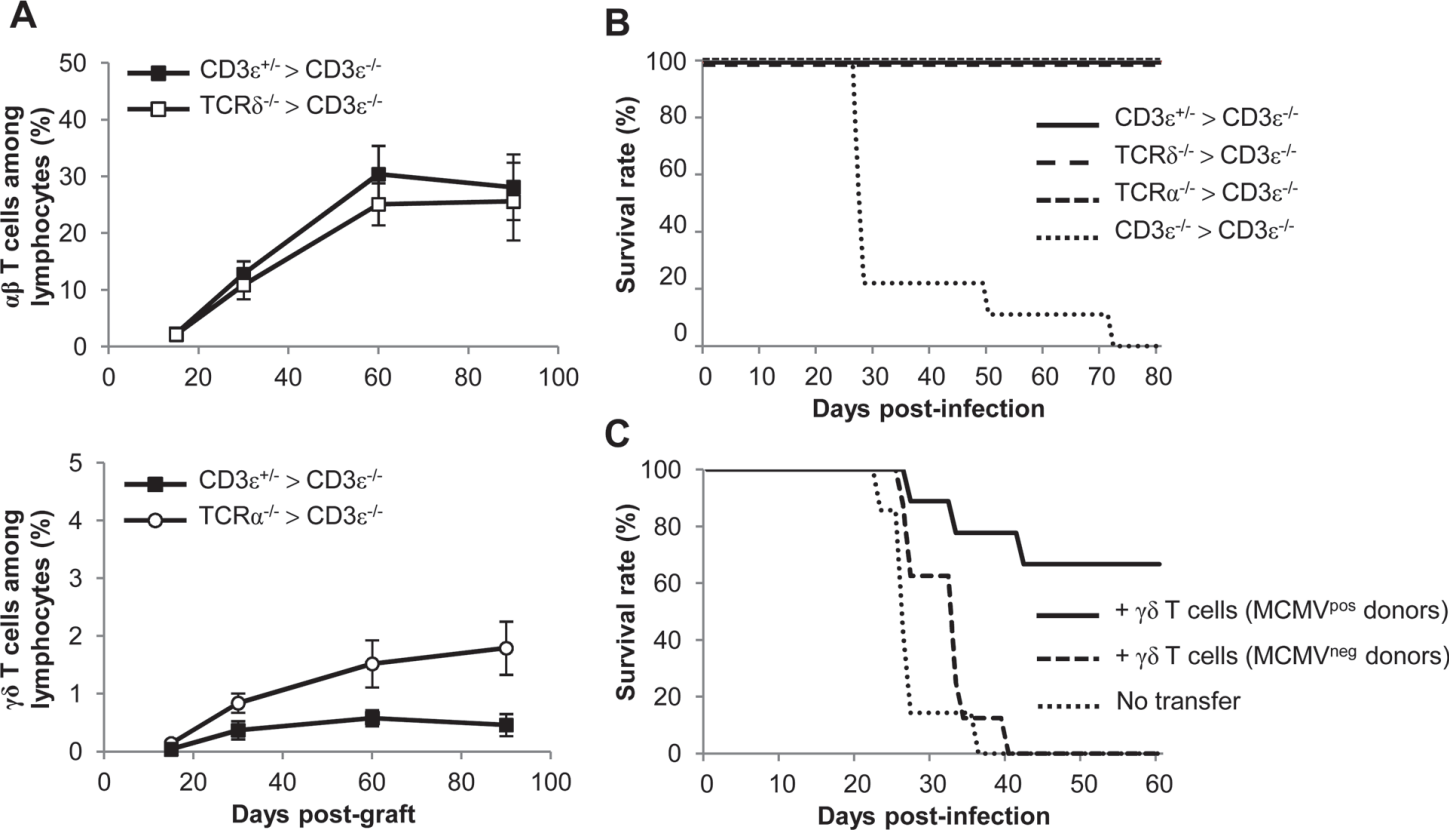
γδ T cell recovery rescues CD3ε^−/−^ mice from MCMV-induced death. **A.** Bone marrows (BM) from TCRδ^−/−^, TCRα^−/−^, CD3ε^+/−^ and CD3ε^−/−^ mice (10 of each) were transferred into CD3ε^−/−^ recipient mice at day 0 (1 donor BM/recipient). At days 15, 30, 60 and 90, blood samples were collected (5 for each grafted mouse line) in order to follow αβ/γδ T cell reconstitution in peripheral blood. The evolution of the proportions of αβ T cells in CD3ε^+/−^ > CD3ε^−/−^ and TCRδ^−/−^ > CD3ε^−/−^ mice are shown (top) as well as the evolution of γδ T cells in CD3ε^+/−^ > CD3ε^−/−^ and TCRα^−/−^ > CD3ε^−/−^ mice (bottom). Results are expressed as percentages among peripheral blood lymphocytes ± SD. **B.** Three months post-graft, CD3ε^+/−^ > CD3ε^−/−^, TCRα^−/−^ > CD3ε^−/−^, TCRδ^−/−^ > CD3ε^−/−^ and CD3ε^−/−^ > CD3ε^−/−^ mice (10 of each) were infected i.p. with 2.10^3^ PFU of MCMV and monitored daily for mortality. This experiment was repeated twice with concordant results. **C.** γδ T cells from uninfected or 14-days infected TCRα^−/−^ mice were purified and i.v. transferred (8–9.10^5^ cells, 92–93% purity) into CD3ε^−/−^ mice (8–9 recipients). 24h after transfer, reconstituted CD3ε^−/−^ mice were challenged with 2.10^3^ PFU of MCMV and monitored daily for mortality. 7 untransferred CD3ε^−/−^ were used as controls. This experiment was repeated twice.

γδ T cells development in CD3ε^−/−^ mice was reconstituted by bone marrow (BM) transfer experiments using TCRα^−/−^ mice as donors (referred to as TCRα^−/−^ > CD3ε^−/−^ mice). This method allowed the generation of the BM-derived Vγ1^+^ and Vγ4^+^ γδ T cell subsets that were increased upon MCMV infection. Control BM transplants were also performed with TCRδ^−/−^ donors (TCRδ^−/−^ > CD3ε^−/−^ mice) and with CD3ε^+/−^ donors (CD3ε^+/−^ > CD3ε^−/−^ mice). γδ and/or αβ T cell reconstitution was allowed to establish for 3 months before MCMV infection of the mice. γδ T cell subset percentages were analyzed in blood from live mice throughout reconstitution (Fig. 6A). Two months after grafting, the percentages of blood γδ and/or αβ T cells (among total lymphocytes) had reached a plateau (Fig. 6A). The proportion of peripheral blood γδ T cells in CD3ε^+/−^ > CD3ε^−/−^ mice was lower than that found in TCRα^−/−^ > CD3ε^−/−^ mice (Fig. 6A, lower panel), in accordance with previous findings which showed that γδ T cells in TCRα^−/−^ outnumbered γδ T cells in C57BL/6 mice [30]. When infected with MCMV at 3 months post-graft, TCRα^−/−^ > CD3ε^−/−^ mice survived MCMV infection as efficiently as CD3ε^+/−^ > CD3ε^−/−^ and TCRδ^−/−^ > CD3ε^−/−^ mice, in marked contrast with CD3ε^−/−^ > CD3ε^−/−^ mice (Fig. 6B).

In a second experimental scenario γδ T cells were purified from TCRα^−/−^ splenocytes and injected intravenously (i.v.) into CD3ε^−/−^ hosts one day before MCMV infection. Surprisingly, very low protection was obtained when γδ T cells isolated from control mice were transferred, whereas γδ T cells from MCMV-infected mice conferred good protection (Fig. 6C).

All together our results confirm the protective anti-CMV role of BM-derived γδ T cells, and show that priming of splenic γδ T cells with MCMV in donor mice is necessary for protection against MCMV after their adoptive transfer.

### γδ T cells are not the main producers of IFNγ and cytolytic granules during early acute MCMV infection

We next sought to gain insight into the mechanism by which γδ T cells exert their antiviral function. CD27 expression was shown to segregate γδ T cells into two functional subsets in mice: CD27^+^ γδ T cells being the main producers of the antiviral cytokine IFNγ and CD27^−^ γδ T cells being prone to secrete IL-17A which is not classically considered as important in antiviral responses [31] [32]. To determine which of these subsets respond to CMV, we analyzed their evolution in organs from MCMV-infected mice. As evidenced in S5A Fig., CD27^−^ cells dominated the γδ T cell response in the lungs, while CD27^+^ and CD27^−^ subtypes were roughly equally implicated in the liver. However, IL-17A transcripts were barely detected in these organs (S5B Fig.). By contrast, IFNγ was expressed in both these organs but noticeably peaked as early as day 3, before the rise of γδ T cell numbers and Cδ transcripts (S5B Fig.).

**Fig 7:**
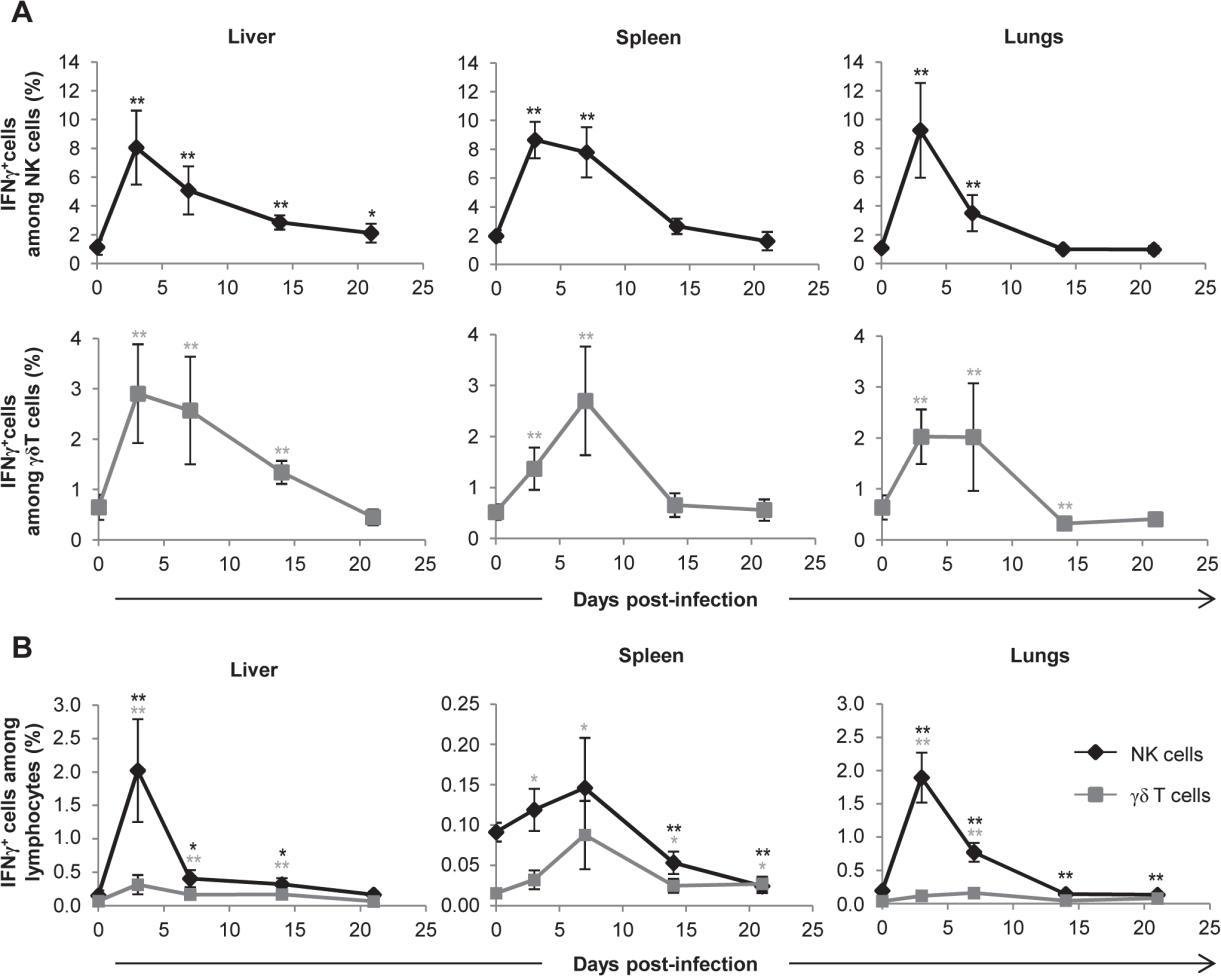
γδ T cells are not the main producers of IFNγ during early acute MCMV infection. TCRα^−/−^ mice were infected i.p. with 2.10^3^ PFU of MCMV. At indicated days post-infection, 6–8 mice were sacrificed and immune cells were isolated from each organ for ex-vivo analysis of IFNγ production by live (7AAD^−^) CD3ε^−^NKp46^+^ and CD3ε^+^γδ^+^ cells. **A**. Proportions of IFNγ producing cells for each NK or γδ T cell subtype are shown. **B**. Percentages of IFNγ producing NK and γδ T cells among lymphocytes. Data are from 1 representative of 2 independent experiments and are expressed as mean percentages ± SEM of 6–8 mice. Statistical differences between day 0 and other time points are shown.

Since the presence of IFNγ transcripts in organs from TCRα^−/−^ infected mice could be attributed to NK cells, we determined IFNγ production at the cellular level by intracellular staining of lymphocytes and using the gating strategy shown in S6 Fig. As shown in Fig. 7A, the proportion of IFNγ-producing NK cells peaked at day 3 in all organs. IFNγ-producing γδ T cells also peaked 3 to 7 days post-infection (Fig. 7A), but represented a minor population when compared to IFNγ-producing NK cells at similar time points (Fig. 7B). Consequently, NK cells were the largely preponderant producers of IFNγ during early acute MCMV infection (Fig. 7B), accounting for 2% of lymphocytes at day 3 in the liver and lungs (i.e. when the relative expression of IFNγ was the highest, S5B Fig.). Similarly, during the course of infection, the proportions of CD107a^+^ NK cells were higher than that of CD107a^+^ γδ T lymphocytes (S7 Fig.). These experiments are in accord with the substantial role of NK cells in the control of early MCMV infection through IFNγ production and cytotoxicity [33], and suggest that the antiviral role of γδ T cells might not principally rely on these two functions.

### NK-independent antiviral protective effect of γδ T cells

**Fig 8:**
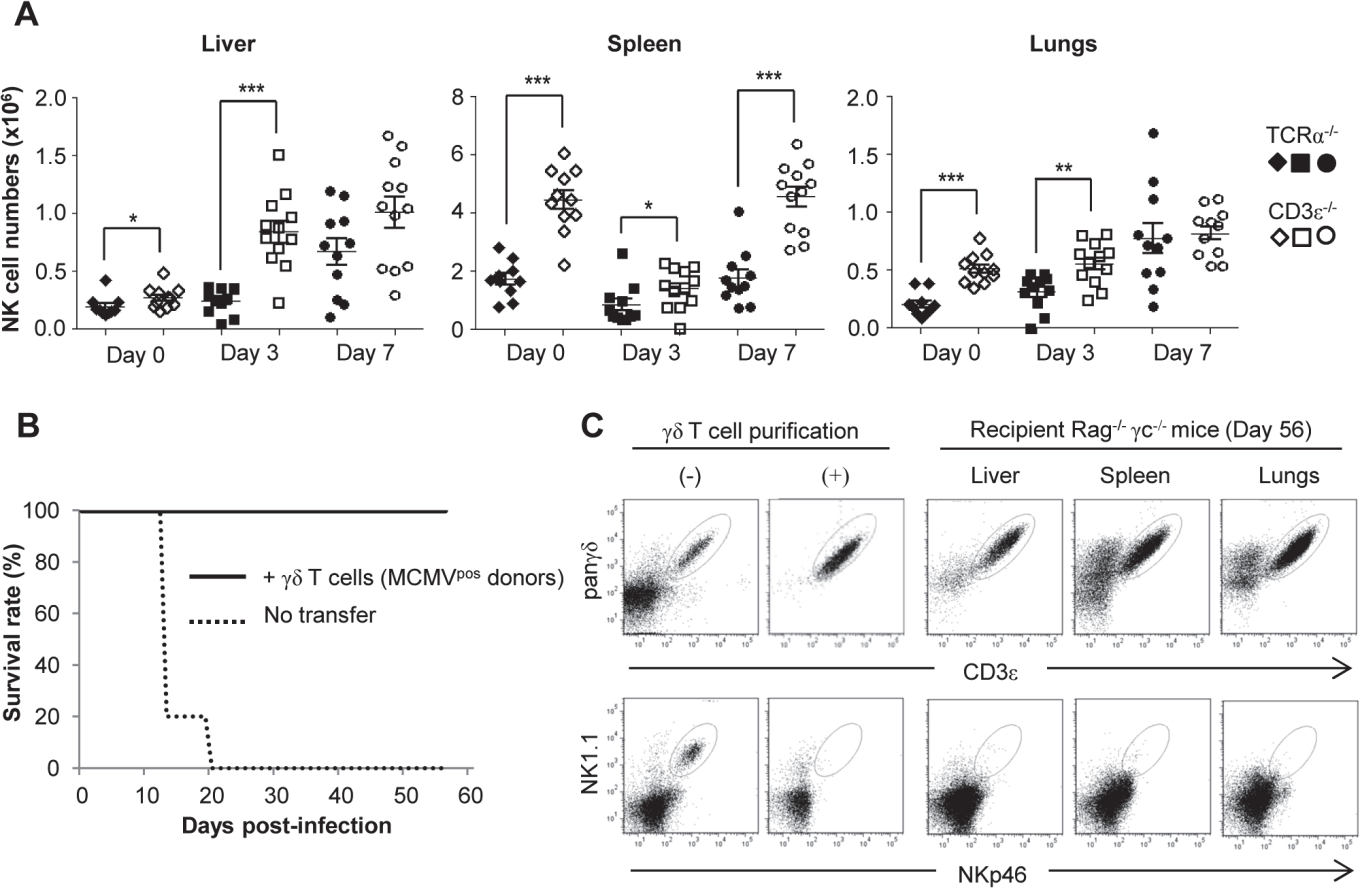
NK-independent antiviral protective effect of γδ T cells. **A.** TCRα^−/−^ and CD3ε^−/−^ mice were uninfected (Day 0) or infected i.p. with 2.10^3^ PFU of MCMV. At indicated days post-infection, 5–6 mice were sacrificed and immune cells were isolated from each organ for flow cytometry analysis. Absolute numbers of NK cells were calculated as described in methods. Black and white symbols represent individual TCRα^−/−^ and CD3ε^−/−^ mice respectively, and horizontal lines represent the mean of 10–12 mice pooled from 2 independent experiments. Differences were evaluated using the Mann-Whitney test: * = p<0,05, ** = p<0,01, *** = p<0,001. **B.** γδ T cells from 14-days infected TCRα^−/−^ mice were purified and i.v. transferred (1.10^6^ cells, 97% purity) into Rag^−/−^γc^−/−^ mice (10 recipients). 24h after transfer, reconstituted Rag^−/−^γc^−/−^ mice were challenged with 2.10^3^ PFU of MCMV and monitored daily for mortality. 5 untransferred Rag^−/−^γc^−/−^ mice were used as controls. Results are from one representative of 2 independent experiments. **C.** Left: flow cytometry analysis of live (7AAD^−^) CD3ε^+^panγδ^+^ T cells (upper panels) and NKp46^+^NK1.1^+^ cells (lower panels) in splenocytes from TCRα^−/−^donors, before (-) and after (+) purification of γδ T cells as described in methods. Right: At day 56 post-infection of Rag^−/−^γc^−/−^ recipients, 3 mice were sacrificed; organs removed and immune cells isolated for flow cytometry analysis of γδ and NK cells. Results are from one representative mouse.

Considering the above results we hypothesized that γδ T cells could exert an indirect antiviral effect by promoting NK cells accumulation as has been previously reported [34]. We therefore compared the evolution of NK cell numbers early post-MCMV infection in TCRα^−/−^ and CD3ε^−/−^ mice. For both mouse lines and as depicted in C57BL/6 wt mice, the overall kinetic was organ-specific with an early decrease of NK cells in the spleen in contrast to liver (Fig. 8A) [35][36]. In contrast to our hypothesis and despite the MCMV-induced death of CD3ε^−/−^ mice, NK cell numbers were globally higher in CD3ε^−/−^ mice than in TCRα^−/−^ mice at all early time points tested (Fig. 8A), showing that γδ T cells antiviral activity was not due to an early increase of NK cells. In addition, when transferred into B/NK/T cells immunodeficient Rag^−/−^γc^−/−^ mice, MCMV-primed γδ T cells were also strikingly sufficient to long term protect these mice from death (Fig. 8B). At day 56, γδ T cells could easily be detected in the liver, spleen and lungs of Rag^−/−^γc^−/−^ recipient mice in contrast to NK cells, demonstrating that the protective function of γδ T cells could act in the total absence of NK cells (Fig. 8C).

## Discussion

Previous work conveys compelling evidence for the implication of human Vδ2^neg^ γδ T cells in the immune response against HCMV infection [5,6,7,9]. However, key questions that cannot easily be addressed in humans remain unanswered, such as the spatial and temporal regulation of the anti-HCMV γδ T cell response and its protective role. Because of its similarity with the human CMV pathogenesis and immune response, the mouse model of MCMV infection has been extensively used and is well characterized. The goal of this study was to take advantage of this model to address these questions concerning the protective role and localization of the γδ T cell response. Herein, we show that γδ T cells are as competent as αβ T cells to protect against CMV challenge, a finding that can be of particular relevance in clinical settings, situations or diseases where αβ T lymphocytes are compromised (hypomorphic Rag1 mutations, individuals treated with immunosuppressive drugs, foetuses or neonates, …) and where γδ T cells have already been shown to expand [6,7,8,9,10,11,12]. This protective function of γδ T cells, under conditions of suboptimal αβ T cell response, has previously been observed earlier in mice in the context of infection by Herpes Simplex Virus type 1 (HSV-1) [37] or by the gut coccidian parasite *Eimeria vermiformis* [38]. These results also corroborate the conserved level of protection against infection observed in patients lacking TCR αβ T cells due to a mutation in the gene coding the TCR α chain [39]. Since γδ T cells have been shown to play an important role in young mice in other infectious models, it would be interesting to evaluate this role in the context of MCMV infection [40]. In addition to extending our results to more a “natural setting” of suboptimal αβ T cells responses, it would allow analysis of the role of non BM-derived γδ T cell subtypes [41]. Finally this MCMV model could be used to evaluate the importance of γδ versus αβ T cells in the context of immunosuppression as used in transplant recipients.

After administration of MCMV via the intraperitoneal route, MCMV targets the liver and spleen as cell free viruses within the first hours before dissemination to the other organs [42]. Accordingly, viral loads were the highest at day 3 in the liver and spleen but peaked at day 7 in the lungs and intestine in all mouse lines tested in the present study. In TCRα^−/−^ mice, viral loads were the lowest at day 14 in the liver and spleen and at day 21 in the lungs (Fig. 2), i.e. after the significant increase of both Vγ1^+^ and Vγ4^+^ γδ T cell subsets in the liver and lungs (Fig. 4A), and of Vγ1^+^ γδ T cells in the spleen (Fig. 5A). Three weeks post-MCMV infection, high viral loads and liver/lung injury were evidenced in CD3ε^−/−^ mice despite normal development and function of NK cells in these mice [28]. In contrast, liver and lung disorders were not observed in TCRα^−/−^ mice at that time. These results are consistent with a role for γδ T cell response/expansion in these organs to control virus multiplication and associated organ damage in the absence of αβ T cells. The protective role of γδ T cells was ascertained by reconstituting γδ T cells in CD3ε^−/−^ mice by bone marrow transplantation, or by adoptive transfer of splenic γδ T cells from TCRα^−/−^ MCMV infected mice. However, when isolated from the spleen of TCRα^−/−^ uninfected mice, γδ T cells were inefficient to induce protection in CD3ε^−/−^ recipients. We can exclude the possibility that lack of protection in CD3ε^−/−^ mice which received naïve γδ T cells was due to an absence of engraftment, because both naïve and MCMV-primed γδ T cells were found in the liver, spleen and lungs of recipient mice (S8 Fig.). The absence of protection by non-primed γδ T cells purified from splenocytes may be due to a delay of reconstitution/differentiation in recipient mice that allow the virus to overwhelm the γδ T cell response. Infection of donor mice by CMV most likely prime γδ splenocytes to readily respond to CMV once transferred in CD3ε^−/−^ mice, compensating this reconstitution limitation.

The development of the anti-CMV immune response involves a complex network of cells from the innate and adaptive immunity that act sequentially to favor health over disease. Research in mice has paid a lot of attention to the early control of MCMV by NK cells, which are responsible for the enhanced resistance of the C57BL/6 mouse strain when compared to BALBc mice. In C57BL/6 mice, NK cell antiviral activity relies on both perforin and IFNγ-release that control viral loads in the liver, spleen and lungs [33,43]. Our *ex vivo* analysis of lymphocytes from C57BL/6 TCRα^−/−^ infected organs show that the early boost (days 3–7) of IFNγ expression and cytotoxic granule exocytosis is mostly due to NK cells, while γδ T cells participate only modestly to these functions (Fig. 7 and S7 Fig.). Thus, although we cannot exclude that this modest contribution might help in controlling MCMV loads, these results rather raise the possibility that γδ T cells operate either by regulating other immune cells or through the production of unknown antiviral mediators. Strikingly, however, our adoptive transfer experiment into Rag^−/−^γc^−/−^ immunodeficient hosts showed that γδ T cell antiviral protective function can be independent of NK/B/αβ T cells. This emphasizes their efficiency and opens interesting perspectives for their possible manipulation in clinical situations where other immune cells are defective.

The kinetics of γδ T cell response was organ specific, with a progressive increase and accumulation of γδ T cells in the lungs, whereas γδ T cells quickly increased and dropped at day 21 in the liver and spleen (Fig. 4A). The persistence within the lungs of memory γδ T cells contrasts with the transient increase of pulmonary γδ T cells that was observed in other murine infectious contexts [44,45,46]. However it reproduces the persistence of γδ T cell expansion in human blood during HCMV-infection which could result from persistent activation of γδ T cells in chronically infected tissues [5,10]. This suggests that the lungs could be an anatomical site for replication of HCMV and chronic activation of γδ T cells, consistent with the fact that HCMV is frequently found in lungs of solid organ transplant patients where it can induce tissue invasive disease [4].

The γδ T cell response to MCMV implicates bone marrow derived Vγ1^+^ and Vγ4^+^ T cells. It will be interesting in the future to determine whether these subsets play similar functions in the response to MCMV, since evidence for distinct roles of Vγ1^+^ and Vγ4^+^ T cells in the protection and/or pathogenesis during infection of mice has been reported [46,47,48]. The involvement of several subsets in the response to MCMV is in agreement with the implication of diverse Vδ2^neg^ T cell subsets (Vδ1, Vδ3, Vδ5) in the response to HCMV [5]. In contrast to long term HCMV-induced γδ T cells that display a restricted CDR3δ length repertoire [5], the CDR3γ1 and γ4 length repertoire of liver, spleen and lung-derived γδ T cells was equivalent in 14-days MCMV-infected and uninfected TCRα^−/−^ mice (S3 Fig. and S4 Fig.). This could reflect a TCR-independent innate-like response of γδ T cells and/or high frequencies of MCMV-specific γδ T cells already existing in naïve mice. However, we cannot exclude the presence of a shared antigen-recognition motif in CDR3γ of different lengths (as observed for the CDR3δ of T22-specific γδ T cells [49]). The number of CDR3γ1 peaks (4 or 5) confirms previous analysis of CDR3 repertoire in mice [50].

Another interesting question concerns the memory function of γδ T cells during MCMV infection, as recently described for CD44^+^CD27^−^ γδ T cells in mouse models of bacterial infections [51,52]. Adaptive and innate like γδ T cells could both participate to memory, in light of the emerging role for innate cells in this context [53]. Previous contact with HCMV induced a rapid recall expansion of effector memory Vδ2^neg^ γδ T cells, which coincided with better infection resolution of HCMV reactivation in transplant recipients [10]. CMV infection in mice also induces CD44^+^CD62L^−^ effector memory γδ T cells that are maintained and outnumber CD44^+^CD62L^+^ central memory γδ T cells at day 56 in all organs (Fig. 4B and Fig. 4C). By definition, effector memory cells are prone to exert rapid functions at the aggression site and the results shown here support the hypothesis that peripheral blood effector-memory human Vδ2^neg^ γδ T cells are re-circulating cells that originate from CMV-targeted organs. It remains to be investigated whether murine γδ T cells recognize self-encoded stress-regulated antigens on CMV-infected cells, as demonstrated for human γδ T cells [18].

Acute infections with HCMV can result in serious disease in infected neonates and in the context of immunosuppression linked to transplantation. Inducing or enhancing the antiviral response of γδ T cells in this context is an attractive objective. Our findings open new perspectives for the use of the murine model of MCMV infection to define the precise mechanism of antiviral activity of γδ T cells and to develop new strategies to induce their activation *in vivo*. Their absence of MHC restriction, their combination of conventional adaptive and innate-like responses, their particular anatomical localization and their dual reactivity against infected and tumor cells, are specific features that place γδ T cells as unique effectors for clinical manipulation. In conjunction with the identification of stress antigens recognized by γδ T cells on infected cells, these results open new avenues for clinical manipulation of γδ T cells against CMV-mediated disease.

## Materials and Methods

### Ethics statement

All experimental procedures involving animals were conducted according to European Union guidelines (European Directive 2010/63/UE) (http://ec.europa.eu/environment/chemical​s/lab_animals/home_en.htm) and approved by the local ethics committee: *Comité d'éthique pour l'expérimentation animale de Bordeaux* (CE50), [project n° 50120197-A].

### Mice

We used C57BL/6 mice. CD3ε^−/−^ [54], TCRα^−/−^ [30] and Rag^−/−^γc^−/−^ mice [55] were from the CDTA (Centre de Distribution, Typage et Archivage Animal, Orléans, France). TCRδ^−/−^ [56] were a gift from Dr Malissen (Centre d’Immunologie de Marseille Luminy, France). Mice were used between 8–12 weeks of age and kept under pathogen-free conditions (Animalerie spécialisée, Université Bordeaux Segalen, France). CD3ε^−/−^ were bred to C57BL/6 mice (C57BL/6J, Charles Rivers laboratory, Larbresle, France) to obtain CD3ε^+/−^ control mice. MCMV-infection was performed in an appropriate animal facility (Animalerie A2, Université Bordeaux Segalen, France).

### Virus stock and infection of mice

MCMV was acquired from the American Type Culture Collection (Smith strain, ATCC VR-194) and propagated into BALBc mice (BALBcBy/J, Charles Rivers laboratory, Larbresle, France) to generate MCMV salivary gland extracts. Virus titers were defined by standard plaque assay on monolayers of mouse embryonic fibroblasts (MEF). Unless indicated, infections were performed by i.p. administration of 2.10^3^ PFU of the salivary gland viral stock.

### AST and ALT quantifications

Mice were bled via the retroorbital sinus after anesthesia (one eye every other week) and the serums collected and frozen. AST and ALT were quantified using standard enzymological methods (laboratoire de Biochimie, CHU Bordeaux, France).

### Tissue processing and histology

Mice were euthanized by cervical dislocation. Liver and lungs were removed, fixed for 24 h in 3.7% neutral-buffered formalin (Sigma-Aldrich), followed by standard histological processing and paraffin embedding. Sections of 4 μm thickness were processed for Hematoxylin/Eosin/Safran (HES) staining (following standard protocols).

### Quantification of MCMV loads

Genomic DNA was isolated from organs using Nucleospin tissue purification kit (Macherey Nagel). Real time PCR to quantify MCMV was performed in Step one plus thermocycler (Applied biosystem) using GoTaq qPCR Master Mix (Promega) with primers specific for MCMV glycoprotein B (gB) (gi330510, forward primer: AGGCCGGTCGAGTACTTCTT and reverse primer: GCGCGGAGTATCAATAGAGC). Known quantities of plasmid comprising MCMV gB were used for the titration curve.

### Relative quantification of transcripts by real time PCR and spectratyping

Total RNA from immune cells was prepared with Nucleospin RNAII kit (Macherey Nagel). Goscript reverse transcriptase (Promega) was used to generate cDNA. Real time PCR was performed in CFX 384 (BioRad). The relative expression of transcripts was determined using the *GAPDH* reference gene. For spectratyping analysis, PCR (40 cycles) was performed with Vγ1 and Cγ4 or with Vγ4 and Cγ1 primers, resulting in amplification of the sequences containing the CDR3γ1 or CDR3γ4, respectively. Then a run-off reaction (one cycle) was performed using a fluorescently labeled Jγ4-FAM primer for CDR3γ1 and with a Jγ1-FAM primer for CDR3γ4 (primers sequences from [50]). The labeled reaction products were run on a capillary sequencer (ABI3730xl analyzer) at ImmuneHealth (Gosselies, Belgium). The fluorescence intensity was analyzed using Peak Scanner 1.0 (Applied Biosystems).

List of primer Fw (forward) and Rv (Reverse):

***GAPDH*** (Genbank NM_008084):

Fw 5’-AATGGGGTGAGGCCGGTGCT-3’

Rv 5’-CACCCTTCAAGTGGGCCCCG-3’

***IFNγ*** (NM_008337.3)

Fw: 5’-ACTGGCAAAAGGATGGTGAC-3’

Rv 5’-TGAGCTCATTGAATGCTTGG-3’

***IL17-A*** (NM_010552.3)

Fw 5’-TCATCTGTGTCTCTGATGCTGTT-3’

Rv 5’-TTGGACACGCTGAGCTTTGA-3’

***Cδ*** (X12729.1)

Fw 5’-CTGTGCACTCGACTGACTTTGAACC-3’

Rv 5’-CCCAGCACCGTGAGGGACATC-3’

**CDR3γ1**

Fw Vγγ1 5'-CCGGCAAAAAGCAAAAAAGT-3

Rv Cγ4 5’-AAGGAGACAAAGGTAGGTCCCAGC-3’

Jγ4-FAM 5'-TACGAGCTTTGTCCCTTTG-3'

**CDR3γ4**

Fw Vγ4 5’-CTTGCAACCCCTACCCATAT-3’

Rv Cγ1 5’-CCACCACTCGTTTCTTTAGG-3’

Jγ1-FAM 5'-CTTAGTTCCTTCTGCAAATACC-3’

### Preparation of immune cells from organs and numeration

We used cell strainers to mash the spleens and livers in RPMI-1640 with 8% FBS; red blood cells were lysed with NH_4_Cl. For the liver, immune cells were isolated by centrifugation (2000 rpm, 20 min) over a 40/80% discontinuous Percoll gradient (GE Healthcare). Pulmonary mononuclear cells were isolated as described [57]. Intestinal intraepithelial mononuclear cells were isolated as described elsewhere [58]. Total organ live cells (unstained with Trypan blue) were then counted using a hemocytometer (Malassez chamber). The proportion of γδ T cells (CD3ε^+^panγδ^+^) and NK cells (NK1.1^+^NKp46^+^) among total organ live cells (7AAD^−^) was evaluated by FACS using a large FSC/SSC gate that included all cells but debris. This proportion was then multiplied by total organ cell number to obtain the absolute number of γδ T cells and NK cells.

### Antibodies and flow cytometry

The following monoclonal antibodies were from BD Pharmingen: anti-CD3ε (145–2C11), anti-TCRδ (GL3), anti-CD44 (IM7), anti-CD62L (MEL-14), anti-CD27 (LG.3A10), anti-NK1.1 (PK136) and anti-NKp46 (29A1.4). Anti-IFNγ (XMG1.2), anti-CD107a (1D4B) and respective isotype control mAbs: Rat IgG1κ (eBRG1) and Rat IgG2aκ (eBR2a) were purchased from eBioscience. Anti-Vγ1 (2.11), anti-Vγ4 (49.2) and anti-Vγ7 (F2.64) mAbs were a kind gift from P. Pereira (Institut Pasteur, Paris). For flow cytometry analysis, immune cells were first incubated with anti-mouse CD16/32 (eBioscience) and stained with relevant antibodies and 7-AAD (BD Pharmingen). Fixed cells were acquired using a LSRFortessa (BD Biosciences), and analyzed using FlowJo software (Tree Star). For intracellular IFNγ staining, cells were incubated in complete medium for 2h at 37°C; 10μg/ml of Brefeldin A (Sigma-Aldrich) was added during the last hour. Intracellular staining was performed after cell surface staining, using BD Cytofix/Cytoperm Fixation/Permeabilization Kit and according to the manufacturer’s instruction (BD Biosciences). For CD107a staining, cells were incubated in complete medium for 2h at 37°C; 10μg/ml Brefeldin A (Sigma-Aldrich) and anti-CD107a or isotype control mAb were added during the last hour. Cells were then stained with relevant monoclonal antibodies.

### Bone marrow transplant experiments

Mice femora and tibia from CD3ε^+/−^, TCRα^−/−^, TCRδ^−/−^ and CD3ε^−/−^ were isolated and the BM was flushed with 1 ml of IMDM with FBS (1%). BM cells from one donor were injected to one CD3ε^−/−^ mice (8–10 per group), intravenously (i.v.) through the retrobulbar sinus in a volume of 0.2 mL IMDM. Mice were conditioned by i.p. injections of Busulfan 22.5 mg/kg (Pierre Fabre laboratory) two days and one day prior to transplantation [59].

### Adoptive transfer experiments

10 TCRα^−/−^ mice were uninfected, or 14 days infected with 2.10^3^ PFU of MCMV. Immune cells were prepared from spleens and pooled before γδ T cell sorting using the TCRγ/δ^+^ T Cell Isolation kit (Miltenyi Biotec). Purity was verified by flow cytometry and 8.10^5^ to 1.10^6^ γδ T cells i.v. transferred into CD3ε^−/−^ or Rag^−/−^γc^−/−^ recipients. 24h after γδ T cell transfer, recipient mice were infected i.p. with 2.10^3^ PFU of MCMV and followed daily. 2–3 months after infection, recipient mice were sacrificed to verify the presence of γδ/NK cells in organs.

### Statistical analysis

Differences were evaluated by the Mann-Whitney test and represented as follows: * = p<0.05, ** = p<0.01, *** = p<0.001, **** = p<0.0001.

## Supporting Information

S1 Fig Longitudinal follow-up of viral loads. TCRδ^−/−^, TCRα^−/−^, CD3ε^+/−^ and CD3ε^−/−^ mice were infected i.p. with 2.10^3^ PFU of MCMV. At indicated days post-infection, 4 mice of each mouse line were dissected and MCMV gB was quantified in organs as described in methods. The experiment was repeated 3 times under similar conditions. Results of 3 independent experiments are depicted as mean of 4 mice for each experiment. Statistical differences between day 3 and other time points are shown.
(TIF)

S2 Fig Gating strategy for flow cytometry analysis of γδ T cells. TCRα^−/−^ mice were infected i.p. with 2.10^3^ PFU of MCMV and sacrificed at different time points. Immune cells were isolated from each organ and stained with indicated antibodies. Lymphoid cells were gated on forward and side scatters (P_1_) and 7-AAD^neg^ viable cells (P_2_) were selected for the analysis of CD3ε^+^panγδ^+^ T cells (P_3_). P_3_ was used for subsequent analysis of Vγ1 and Vγ4 (or Vγ7) expression. Data are from one representative mouse.
(TIF)

S3 Fig The CDR3γ1 repertoire of liver-, spleen- and lung-derived γδ T cells does not change upon MCMV infection as assessed by spectratyping. Mice (6 of each) were uninfected (Day 0) or infected 14 days with 2.10^3^ PFU of MCMV. The liver, spleen and lungs were removed and the RNA prepared for spectratyping analysis as described in the materials and methods. Each box represents the CDR3γ1 data of one different mouse. Above each box the corresponding mouse ID is indicated.
(TIF)

S4 Fig The CDR3γ4 repertoire of liver-, spleen- and lung-derived γδ T cells does not change upon MCMV infection as assessed by spectratyping. Mice (6 of each) were uninfected (Day 0) or infected 14 days with 2.10^3^ PFU of MCMV. The liver, spleen and lungs were removed and the RNA prepared for spectratyping analysis as described in the materials and methods. Each box represents the CDR3γ4 data of one different mouse. Above each box the corresponding mouse ID is indicated.
(TIF)

S5 Fig γδ T cells are not the main producers of IFNγ and cytolytic granules during early acute MCMV infection. TCRα^−/−^ mice were infected i.p. with 2.10^3^ PFU of MCMV. At indicated days post-infection, 5–9 mice were sacrificed and immune cells were prepared from each organ. **A.** Kinetics of absolute CD27^+^ and CD27^−^ γδ T cell numbers. The proportions of CD27^+^ and CD27^−^ γδ T cells among live cells were determined by flow cytometry analysis and reported to total organ cell counts. **B.** Total RNA was prepared and transcripts for indicated molecules were quantified as described in methods. These experiments were performed twice with comparable results and data are the means ± SEM of 8–9 mice from one experiment. Statistical differences between day 0 and other time points are shown.
(TIF)

S6 Fig Gating strategy for flow cytometry analysis of IFNγ producing γδ T cells and NK cells. TCRα^−/−^ mice were infected i.p. with 2.10^3^ PFU of MCMV and sacrificed at different time points. Immune cells were isolated from each organ and stained with indicated antibodies. Lymphoid cells were gated on forward and side scatters (P_1_) and 7-AAD^−^ viable cells (P_2_) were selected for the analysis of CD3ε^+^panγδ^+^ T cells (P_3_) and CD3ε^−^NKp46^+^ cells (P_5_). IFNγ-producing γδ T cells (P_4_) were analysed among total γδ T cells (P_3_) or among live lymphocytes (P_2_). IFNγ-producing NK cells (P_6_) were analysed among total NK cells (P_5_) or among live lymphocytes (P_2_). Data are from the liver of one representative mouse.
(TIF)

S7 Fig γδ T cells are not the main cytotoxic effectors during acute MCMV infection TCRα^−/−^ mice were infected i.p. with 2.10^3^ PFU of MCMV. At indicated days post-infection, 6–8 mice were sacrificed and immune cells were prepared from each organ for flow cytometry analysis. The proportions of CD107a^+^ for each CD3ε^−^NKp46^+^ (NK) or CD3ε^+^γδ^+^ (γδ) cell subtype are shown, as well as percentages of CD107a^+^ NK and CD107a^+^ γδ T cells among lymphocytes. Data are from 1 representative of 2 independent experiments and are expressed as the mean percentages ± SEM of 6–8 mice. Statistical differences between day 0 and other time points are indicated.
(TIF)

S8 Fig γδ T cells are present in the liver, spleen and lungs of adoptively transferred mice. γδ T cells from uninfected or 14-days infected TCRα^−/−^ mice were purified and i.v. transferred (8–9.10^5^ cells, 92–93% purity) into CD3ε^−/−^ mice (8–9 recipients). 24h after transfer, reconstituted CD3ε^−/−^ mice were challenged with 2.10^3^ PFU of MCMV and monitored daily for mortality. 3 naïve γδ T cells transferred mice were sacrificed at day 26 just before death (anticipated by defined signs of infection such as piloerection) and all MCMV-primed γδ T cells transferred mice were sacrificed at day 62 (end of the experiment). Immune cells were prepared from liver, spleen and lungs for flow cytometry analysis of live (7AAD^−^) CD3ε^+^γδ^+^ cells. Data are from one representative mouse for each group.
(TIF)
