# Supplementary figures and images for: γδ T Cells Confer Protection against Murine Cytomegalovirus (MCMV)
Source: PLoS Pathog. 2015 Mar 6;11(3):e1004702. doi: 10.1371/journal.ppat.1004702 (PMC4352080; doi:10.1371/journal.ppat.1004702)

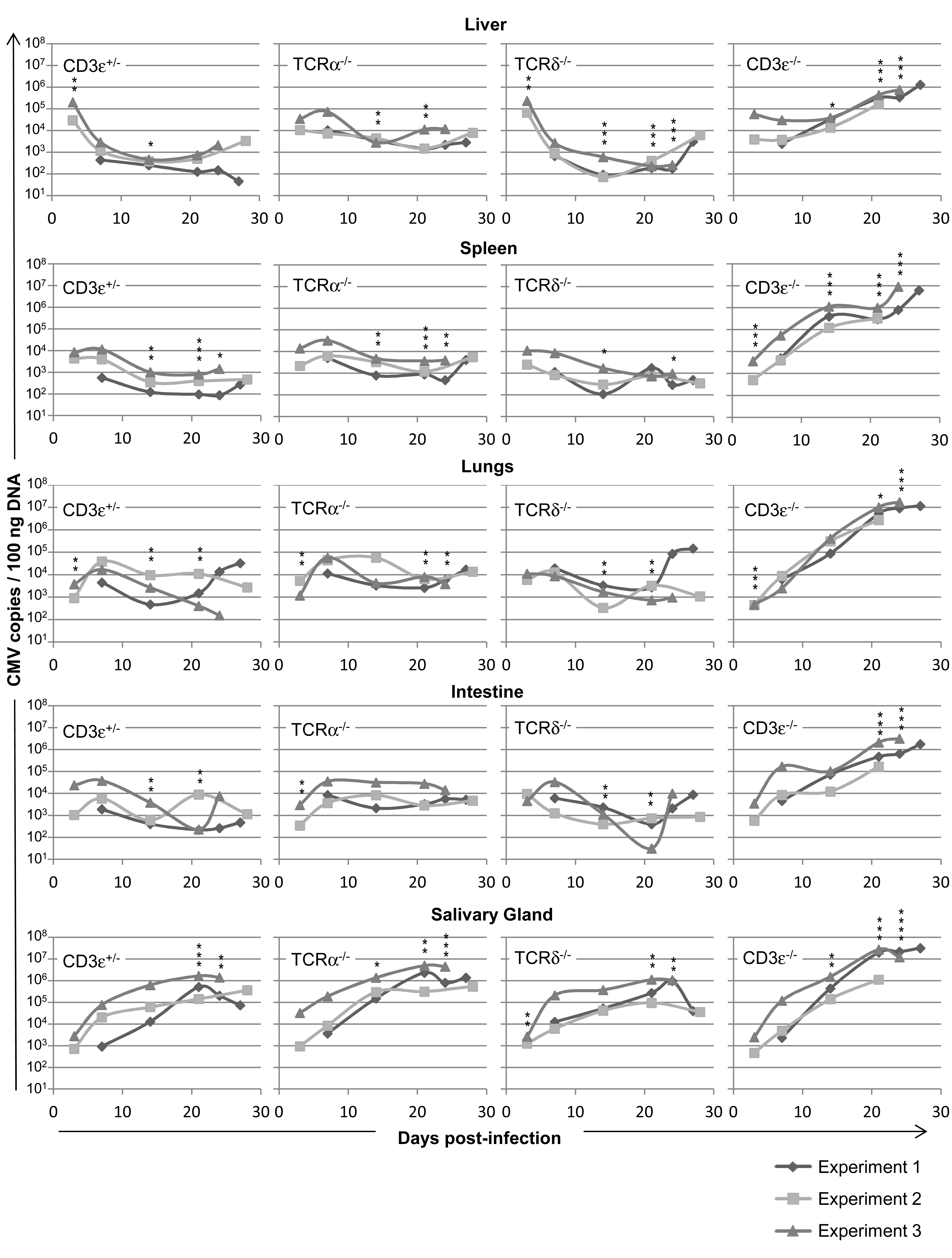

Supplement: S1 Fig — TCRδ−/−, TCRα−/−, CD3ε+/− and CD3ε−/− mice were infected i.p. with 2.103 PFU of MCMV. At indicated days post-infection, 4 mice of each mouse line were dissected and MCMV gB was quantified in organs as described in methods. The experiment was repeated 3 times under similar conditions. Results of 3 independent experiments are depicted as mean of 4 mice for each experiment. Statistical differences between day 3 and other time points are shown. (TIF) [file ppat.1004702.s001.tif]

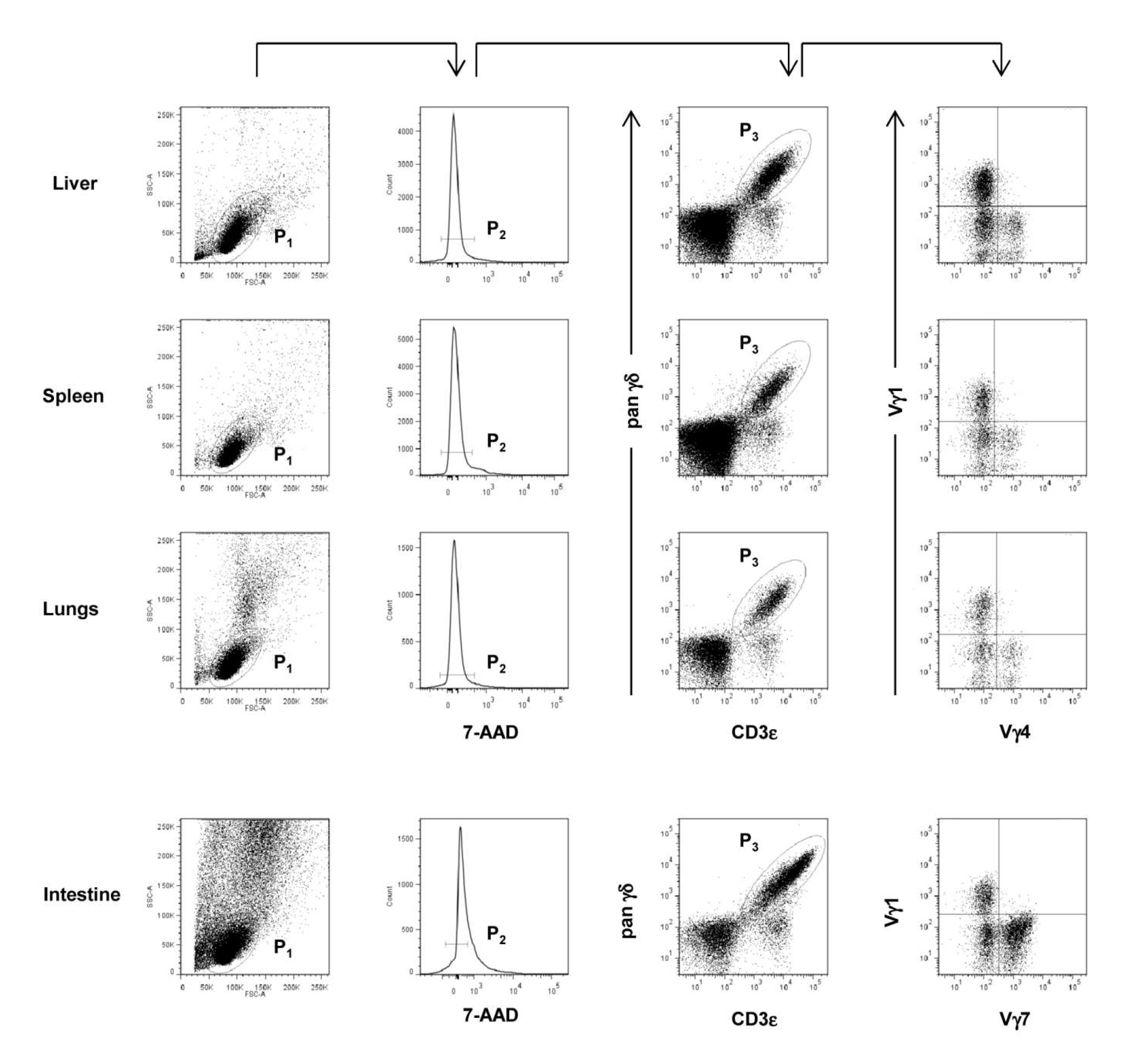

Supplement: S2 Fig — TCRα−/− mice were infected i.p. with 2.103 PFU of MCMV and sacrificed at different time points. Immune cells were isolated from each organ and stained with indicated antibodies. Lymphoid cells were gated on forward and side scatters (P1) and 7-AADneg viable cells (P2) were selected for the analysis of CD3ε+panγδ+ T cells (P3). P3 was used for subsequent analysis of Vγ1 and Vγ4 (or Vγ7) expression. Data are from one representative mouse. (TIF) [file ppat.1004702.s002.tif]

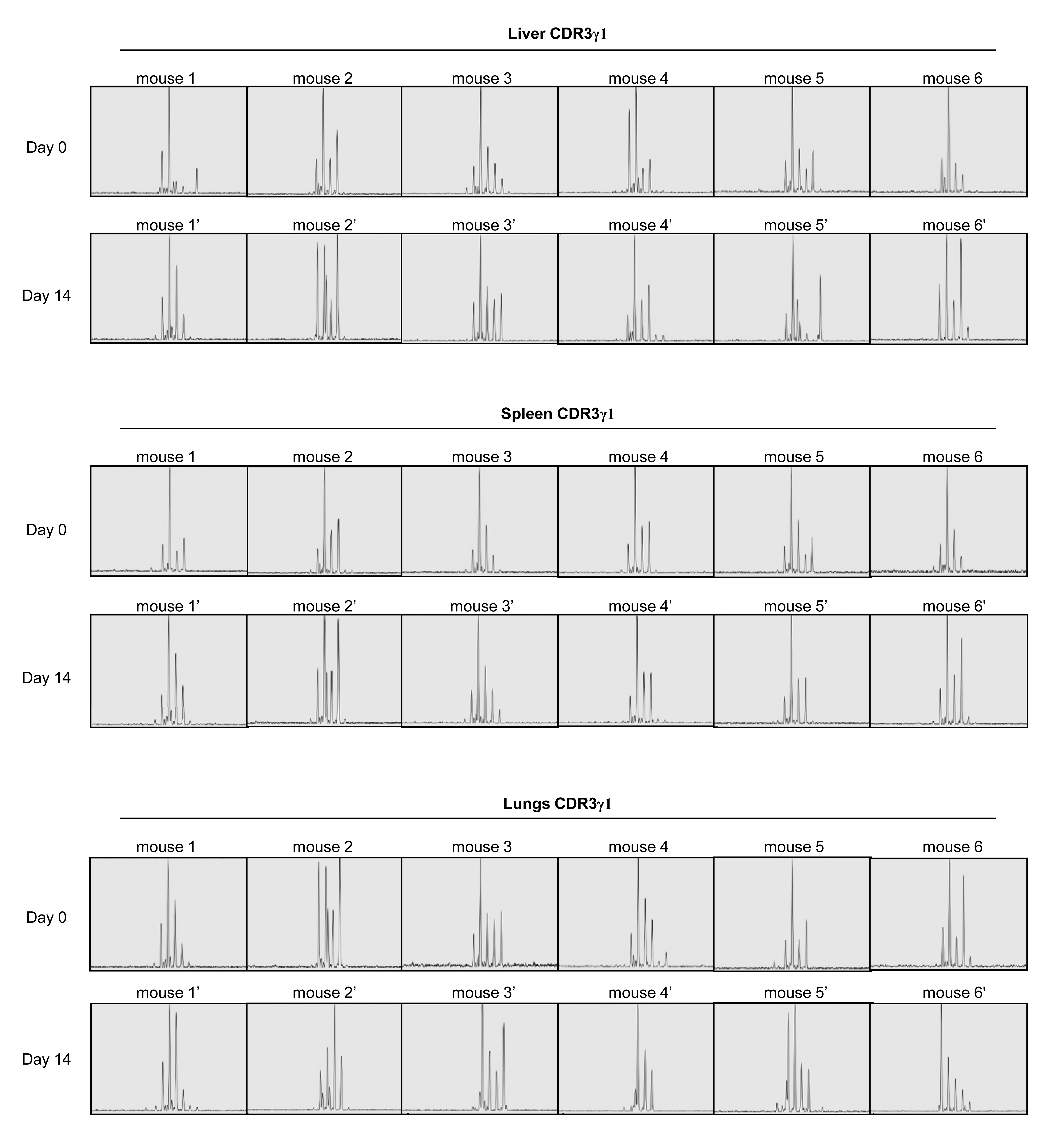

Supplement: S3 Fig — Mice (6 of each) were uninfected (Day 0) or infected 14 days with 2.103 PFU of MCMV. The liver, spleen and lungs were removed and the RNA prepared for spectratyping analysis as described in the materials and methods. Each box represents the CDR3γ1 data of one different mouse. Above each box the corresponding mouse ID is indicated. (TIF) [file ppat.1004702.s003.tif]

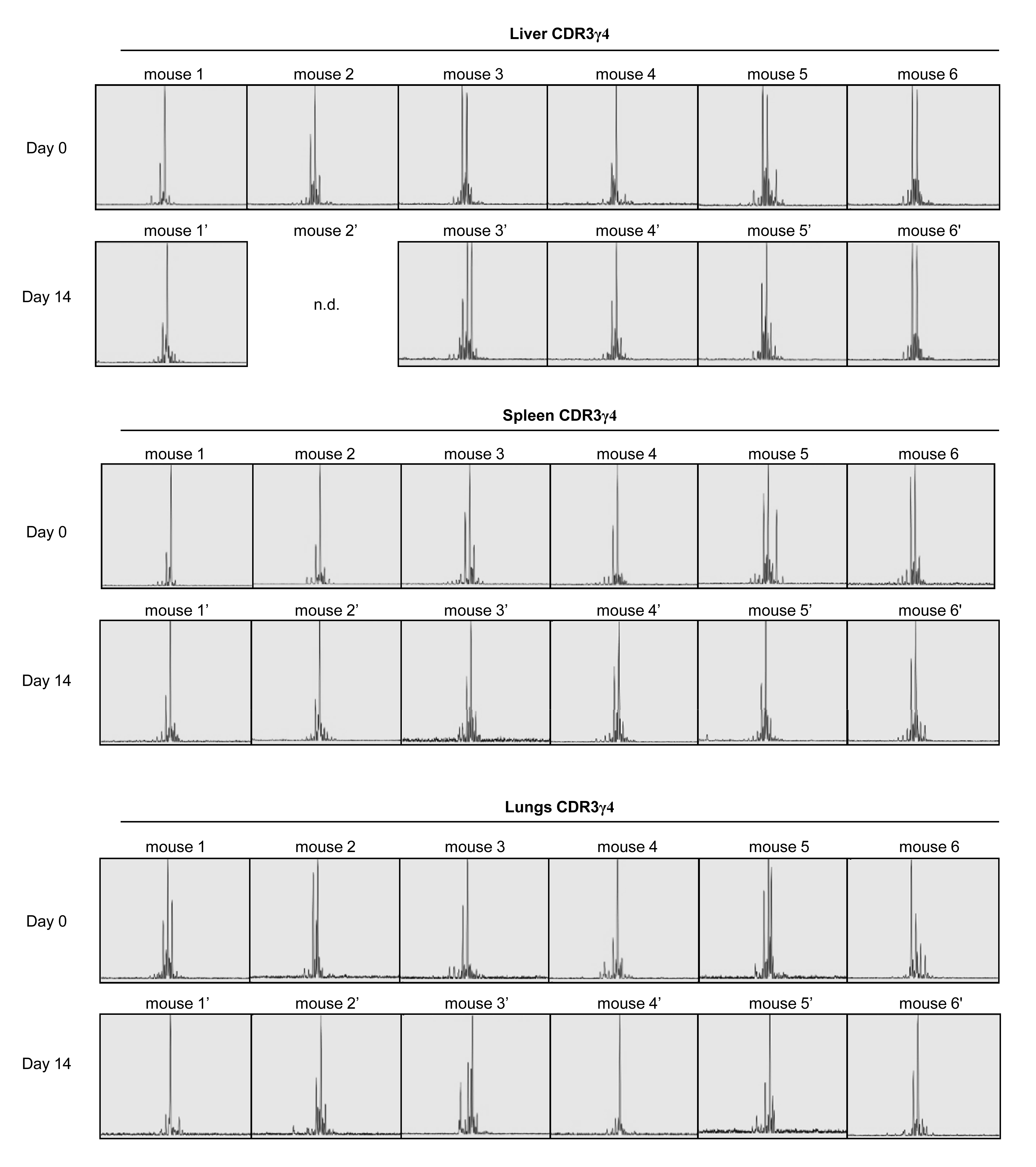

Supplement: S4 Fig — Mice (6 of each) were uninfected (Day 0) or infected 14 days with 2.103 PFU of MCMV. The liver, spleen and lungs were removed and the RNA prepared for spectratyping analysis as described in the materials and methods. Each box represents the CDR3γ4 data of one different mouse. Above each box the corresponding mouse ID is indicated. (TIF) [file ppat.1004702.s004.tif]

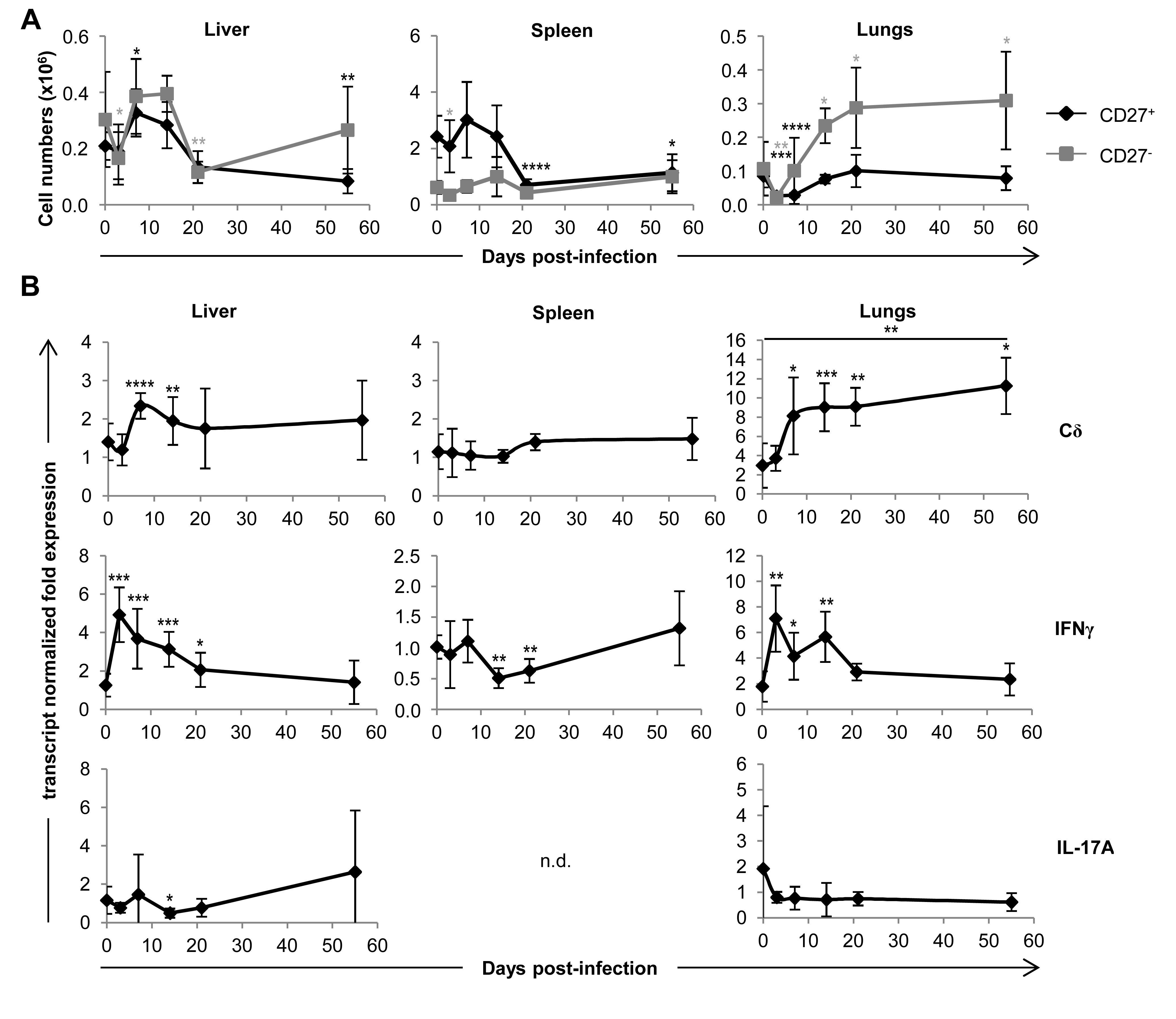

Supplement: S5 Fig — TCRα−/− mice were infected i.p. with 2.103 PFU of MCMV. At indicated days post-infection, 5–9 mice were sacrificed and immune cells were prepared from each organ. A. Kinetics of absolute CD27+ and CD27− γδ T cell numbers. The proportions of CD27+ and CD27− γδ T cells among live cells were determined by flow cytometry analysis and reported to total organ cell counts. B. Total RNA was prepared and transcripts for indicated molecules were quantified as described in methods. These experiments were performed twice with comparable results and data are the means ± SEM of 8–9 mice from one experiment. Statistical differences between day 0 and other time points are shown. (TIF) [file ppat.1004702.s005.tif]

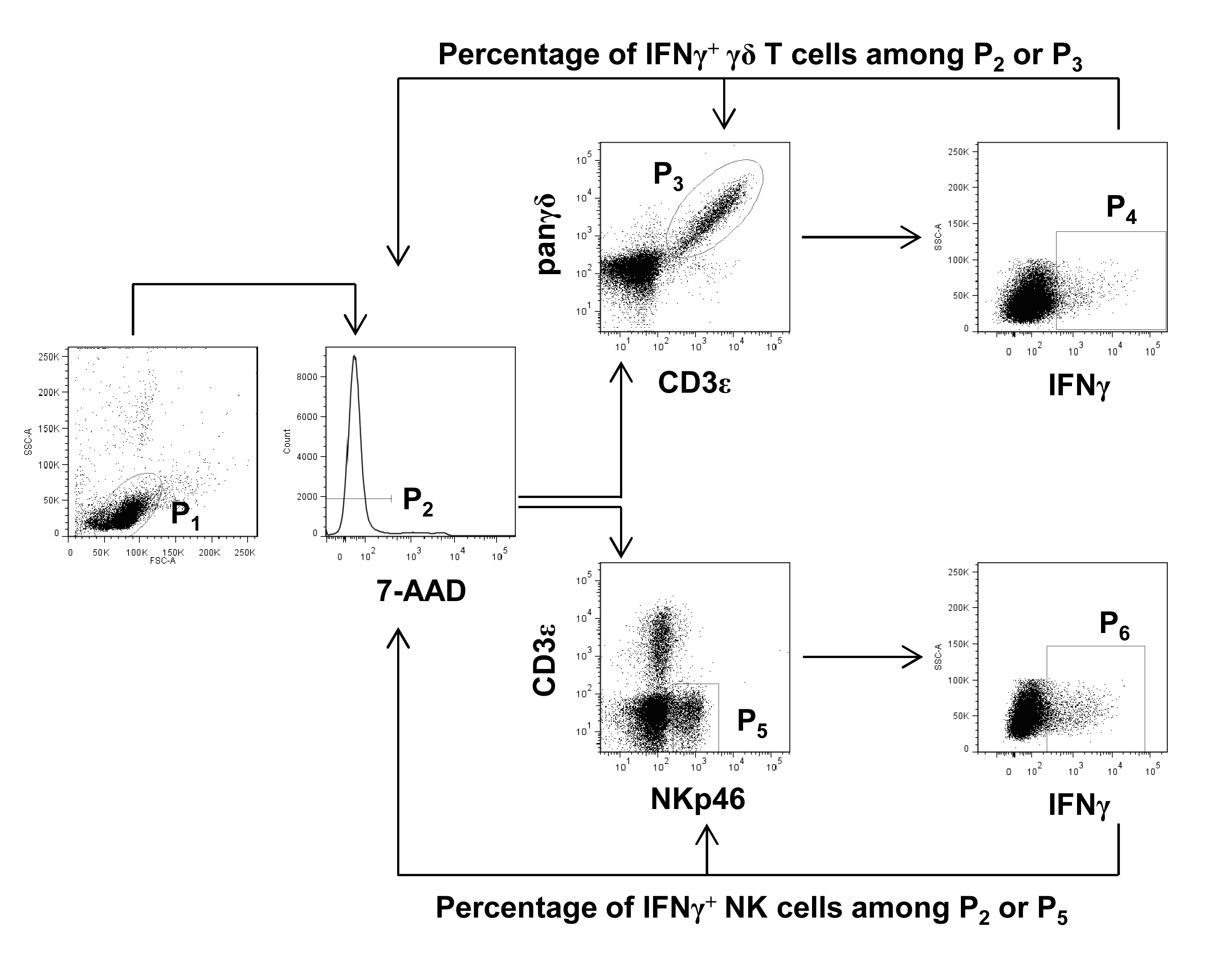

Supplement: S6 Fig — TCRα−/− mice were infected i.p. with 2.103 PFU of MCMV and sacrificed at different time points. Immune cells were isolated from each organ and stained with indicated antibodies. Lymphoid cells were gated on forward and side scatters (P1) and 7-AAD− viable cells (P2) were selected for the analysis of CD3ε+panγδ+ T cells (P3) and CD3ε−NKp46+ cells (P5). IFNγ-producing γδ T cells (P4) were analysed among total γδ T cells (P3) or among live lymphocytes (P2). IFNγ-producing NK cells (P6) were analysed among total NK cells (P5) or among live lymphocytes (P2). Data are from the liver of one representative mouse. (TIF) [file ppat.1004702.s006.tif]

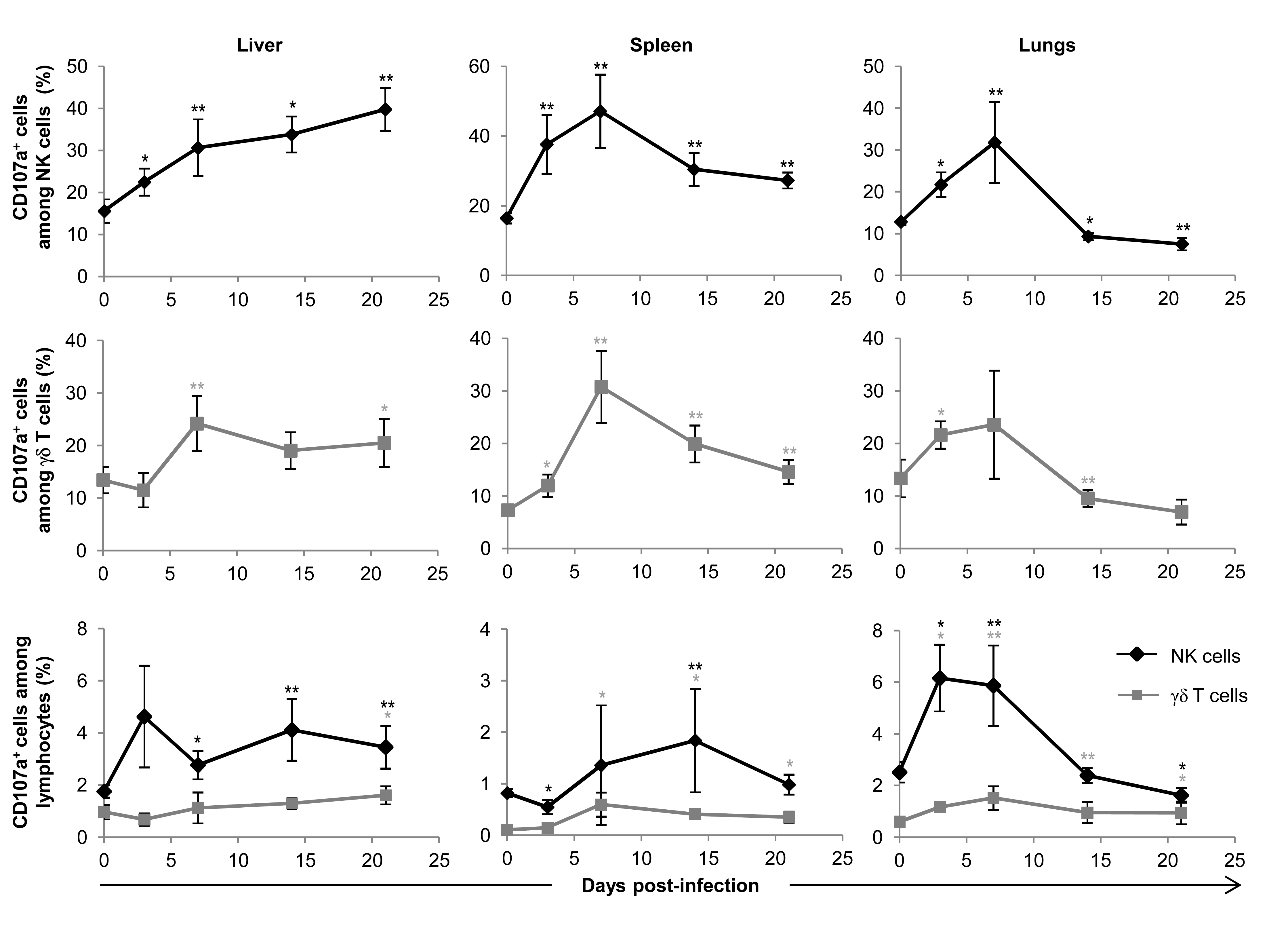

Supplement: S7 Fig — At indicated days post-infection, 6–8 mice were sacrificed and immune cells were prepared from each organ for flow cytometry analysis. The proportions of CD107a+ for each CD3ε−NKp46+ (NK) or CD3ε+γδ+ (γδ) cell subtype are shown, as well as percentages of CD107a+ NK and CD107a+ γδ T cells among lymphocytes. Data are from 1 representative of 2 independent experiments and are expressed as the mean percentages ± SEM of 6–8 mice. Statistical differences between day 0 and other time points are indicated. (TIF) [file ppat.1004702.s007.tif]

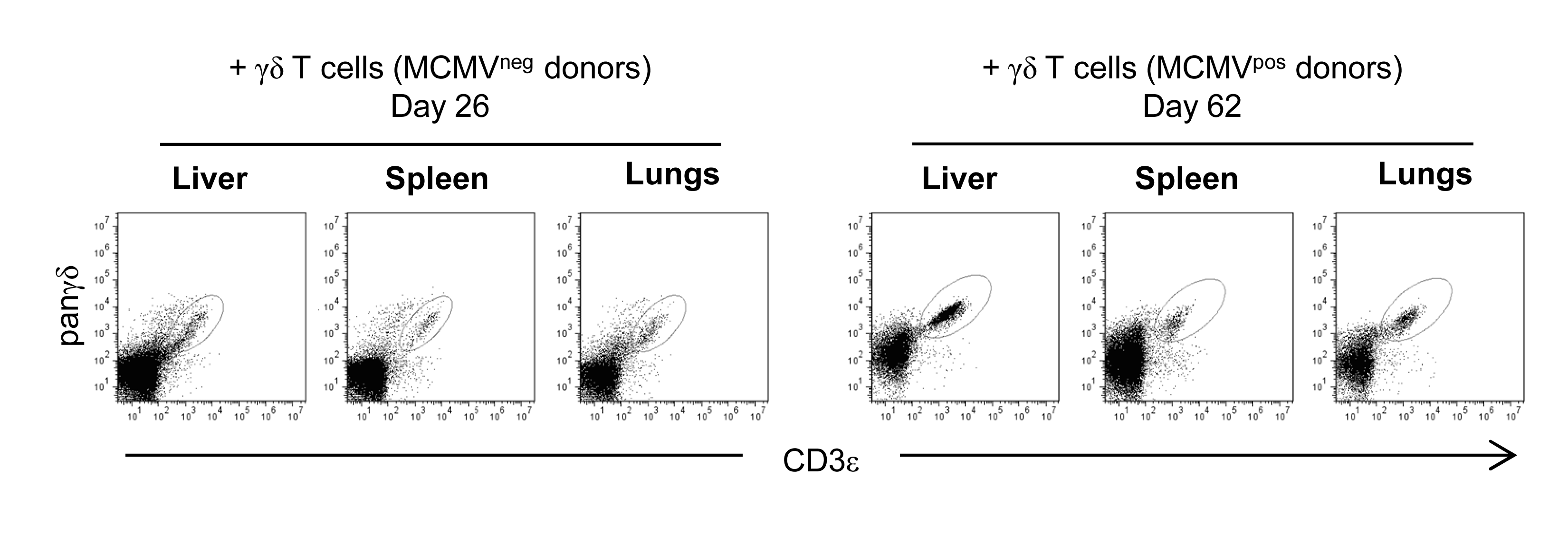

Supplement: S8 Fig — γδ T cells from uninfected or 14-days infected TCRα−/− mice were purified and i.v. transferred (8–9.105 cells, 92–93% purity) into CD3ε−/− mice (8–9 recipients). 24h after transfer, reconstituted CD3ε−/− mice were challenged with 2.103 PFU of MCMV and monitored daily for mortality. 3 naïve γδ T cells transferred mice were sacrificed at day 26 just before death (anticipated by defined signs of infection such as piloerection) and all MCMV-primed γδ T cells transferred mice were sacrificed at day 62 (end of the experiment). Immune cells were prepared from liver, spleen and lungs for flow cytometry analysis of live (7AAD−) CD3ε+γδ+ cells. Data are from one representative mouse for each group. (TIF) [file ppat.1004702.s008.tif]
